# Protein Prenylation and Hsp40 in Thermotolerance of Plasmodium falciparum Malaria Parasites

**DOI:** 10.1128/mBio.00760-21

**Published:** 2021-06-29

**Authors:** Emily S. Mathews, Andrew J. Jezewski, Audrey R. Odom John

**Affiliations:** a Department of Pediatrics, Washington University School of Medicine, St. Louis, Missouri, USA; b Department of Pediatrics, Carver College of Medicine, University of Iowa, Iowa City, Iowa, USA; c Department of Molecular Microbiology, Washington University School of Medicine, St. Louis, Missouri, USA; d Division of Infectious Disease, Department of Pediatrics, Children’s Hospital of Philadelphia, Perelman School of Medicine, University of Pennsylvania, Philadelphia, Pennsylvania, USA; Washington University School of Medicine

**Keywords:** *Plasmodium falciparum*, farnesylation, heat shock, isoprenoids, malaria, protein chaperone

## Abstract

During its complex life cycle, the malaria parasite survives dramatic environmental stresses, including large temperature shifts. Protein prenylation is required during asexual replication of Plasmodium falciparum, and the canonical heat shock protein 40 protein (HSP40; PF3D7_1437900) is posttranslationally modified with a 15-carbon farnesyl isoprenyl group. In other organisms, farnesylation of Hsp40 orthologs controls their localization and function in resisting environmental stress. In this work, we find that plastidial isopentenyl pyrophosphate (IPP) synthesis and protein farnesylation are required for malaria parasite survival after cold and heat shock. Furthermore, loss of HSP40 farnesylation alters its membrane attachment and interaction with proteins in essential pathways in the parasite. Together, this work reveals that farnesylation is essential for parasite survival during temperature stress. Farnesylation of HSP40 may promote thermotolerance by guiding distinct chaperone-client protein interactions.

## INTRODUCTION

Infection with the protozoan parasite Plasmodium falciparum causes the majority of cases of severe and fatal malaria. P. falciparum must recognize and adapt to dramatic environmental stresses, as its complex life cycle requires development in both an invertebrate mosquito vector and the warm-blooded vertebrate human host. In particular, temperature is critical at every stage of the parasite life cycle. In the mosquito vector, many temperature-sensitive factors contribute to human transmission, such as biting rate, vector longevity, parasite development, and vector competence (1). Human infection begins upon the bloodmeal of a female *Anopheles* mosquito. Entering the human host, where the normal physiological temperature is 37°C, sporozoite-stage parasites experience heat shock. However, this temperature stress is necessary for efficient hepatocyte infection and the resulting amplification of infection (2, 3). The parasite emerges from the liver to initiate asexual replication within erythrocytes, the clinically symptomatic stage of *Plasmodium* infection. A pathognomonic feature of falciparum malaria is periodic episodes of fever (to 41°C or more) recurring every 48 h, corresponding to the synchronous rupture of infected erythrocytes and daughter merozoite release (4). In contrast, the sexual-stage parasites that return to the mosquito vector are again exposed to cold temperature shock, as the parasite must now readjust to approximately 25°C. While temperature fluctuations are an inherent part of the malaria life cycle, how the parasite copes with thermal stress is not well understood.

Temperature regulates both malaria pathogenesis and antimalarial sensitivity. Controlled hypothermia (32°C) has been used clinically to improve outcomes of severe cerebral malaria (5). *In vitro*, hypothermia (32°C) inhibits P. falciparum growth (6), and a similar effect occurs at lower temperatures (28°C) (7). While the potency of some antimalarials (e.g., chloroquine, mefloquine, and pyronaridine) are unaffected by lower temperatures (6, 8), susceptibility to artemisinin—the backbone of front-line artemisinin-based combination therapies—is modulated by both cold and heat stresses (6, 8). As temperature fluctuations are an inherent part of the P. falciparum life cycle, this common environmental stress may affect the ability of antimalarials to influence essential parasite targets. Antimalarial resistance threatens malaria control efforts worldwide. In particular, rising rates of delayed clearance to artemisinin-based combination therapies, including resistance to artemisinin partner drugs, has raised concerns about emerging multidrug resistance (9–17). Thus, there is a pressing need to identify essential survival pathways in P. falciparum, such as thermotolerance, in order to support ongoing development of new antimalarial agents.

During intraerythrocytic development, P. falciparum assembles isoprenoids *de novo* through the methylerythritol 4-phosphate (MEP) pathway (18–20), localized within the unusual plastidial organelle of the parasite, the apicoplast. Chemical inhibition of this pathway by the small molecule fosmidomycin (FSM) is lethal to malaria parasites (18). FSM-mediated growth inhibition can be rescued by supplementation with isoprenoids such as isopentenyl pyrophosphate (IPP) (21, 22). These studies validate the essentiality of isoprenoid synthesis in asexual P. falciparum, but there have been long-standing questions regarding which biological processes in the parasite require apicoplast isoprenoid biosynthesis. Protein prenylation appears to be a core essential function of isoprenoid biosynthesis in malaria parasites (23–27). During protein prenylation, either a farnesyl (FPP; 15-carbon) or a geranylgeranyl (GGPP; 20-carbon) isoprenyl group is posttranslationally attached to C-terminal cysteines by one of three well-characterized prenyltransferases, farnesyltransferase (FTase) and geranylgeranyltransferases type I and type II (GGTase I, GGTase II). Chemical inhibition of prenyltransferases with small molecules (e.g., FTase inhibitor FTI-277) inhibits parasite growth (24–29), providing compelling evidence that prenylated malarial proteins and their unidentified downstream biological processes include potential antimalarial targets. We and others have used chemical labeling to characterize the complete prenylated proteome of intraerythrocytic P. falciparum (30, 31). These studies identify a single heat shock protein 40 (HSP40; PF3D7_1437900) as robustly farnesylated during intraerythrocytic replication.

Heat shock proteins are necessary for protein folding and stabilization. Importantly, heat shock proteins play a vital role in surviving cellular stresses that might otherwise be lethal, and therefore heat shock protein expression is upregulated under diverse cellular insults, including heat and cold shock. The main functions of Hsp40 family members are to identify and bind partially misfolded proteins in order to initiate Hsp70-mediated refolding. Inhibition of Hsp70 in P. falciparum was recently shown to hypersensitize parasites to heat shock conditions (32). As heat shock proteins have a known role in temperature-dependent survival, it is perhaps unsurprising that roughly 2% of the P. falciparum genome is dedicated to molecular chaperones, including a large number of heat shock proteins (33). HSP40 is a member of an expanded Hsp40 family in P. falciparum comprising 49 total members (34). The majority of Hsp40 family members in P. falciparum are unique and not shared with other Apicomplexa (34, 35). HSP40 is predicted to be the only canonical Hsp40 and the main cochaperone of Hsp70 in P. falciparum because of its similar heat inducibility and localization (36). The lack of additional canonical Hsp40s in P. falciparum suggests that HSP40 is necessary for parasite development. Critically, HSP40 is the sole prenylated heat shock protein in P. falciparum (30, 31). In yeast, prenylation of the HSP40 homolog YDJ1 is required for thermotolerance, protein localization, and interaction with client proteins (37–39). In P. falciparum, the role of farnesylated HSP40 (farnesyl-HSP40) has not previously been investigated.

In this study, we investigate the role of the apicoplast MEP pathway of isoprenoid biosynthesis and downstream protein prenylation on survival during environmental stress in P. falciparum. We find that plastidial IPP production is critical to parasite survival following either heat (40°C) or cold shock (25°C). In addition, we find that farnesylation, but not geranylgeranylation, is required for thermotolerance in P. falciparum. We also demonstrate that the farnesylated heat shock protein HSP40 is likely essential. Farnesylation of HSP40 mediates its membrane association and directs its interaction with proteins in essential pathways in the parasite. Our work suggests HSP40 prenylation as a compelling candidate for a downstream effector by which IPP synthesis and protein farnesylation contribute to parasite stress survival.

## RESULTS

### Thermotolerance in malaria parasites requires IPP synthesis and protein farnesylation.

The malaria parasite must adapt to diverse environmental stresses, such as large temperature shifts, throughout its complex life cycle. Heat shock proteins play an important role in the ability of the parasite to survive temperature stress (40–43). Because P. falciparum expresses a farnesylated heat shock protein, HSP40, we hypothesized that production of isoprenoids and protein prenylation are required for growth during temperature stress. We tested this hypothesis by inhibiting protein prenylation and applying either heat or cold stress.

**FIG 1:**
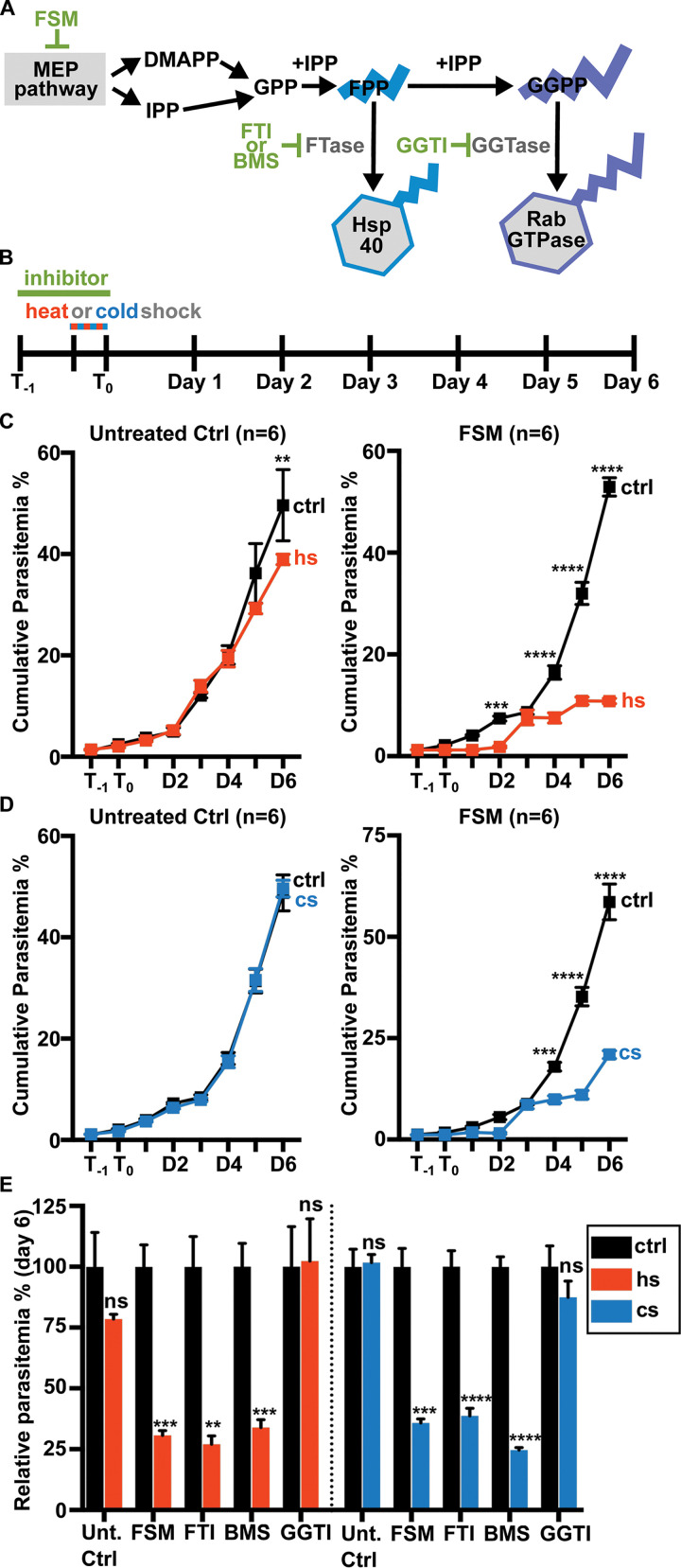
Growth under temperature stress requires IPP synthesis and protein farnesylation. (A) Prenylphosphate substrates for protein prenylation are derived from the nonmevalonate MEP pathway. MEP pathway products, IPP and dimethylallyl pyrophosphate (DMAPP), serve as precursors to FPP used by FTase and GGPP used by GGTase in protein prenylation. FSM treatment inhibits production of IPP and DMAPP. Farnesyltransferase inhibitors (FTI or BMS) inhibit protein farnesylation, while geranylgeranyltransferase inhibitors (GGTI) prevent protein geranylgeranylation. (B) Parasites were treated with FSM (5 μM), farnesyltransferase inhibitors (FTI [10 μM] and BMS [200 nM]), or geranylgeranyltransferase inhibitor GGTI (2 μM) for 24 h prior to a 6-h heat (40°C) or cold (25°C) shock. (C and D) FSM-treated parasite growth is significantly reduced after heat shock (C) and cold shock (D). (E) Inhibition of farnesylation by treating parasites with FTI or BMS significantly reduced growth after temperature stress. Growth in GGTI-treated parasites is unchanged after heat or cold shock. (C to E) *n* = 6; **, *P* ≤ 0.01; ***, *P* ≤ 0.001; ****, *P* ≤ 0.0001. (C and D) 2-way ANOVA, *P* values adjusted for multiple comparisons using Sidak’s multiple-comparison test. (E) Within each treatment group, the normalized control was compared to temperature shock sample by unpaired *t* test with Welch’s correction. Abbreviations: ctrl, control, hs, heat shock, cs, cold shock.

Chemically diverse small molecule inhibitors affect protein prenylation during asexual replication of *Plasmodium* spp. For example, treatment with FSM, which inhibits upstream isoprenoid biosynthesis and therefore synthesis of prenylphosphates, reduces downstream protein prenylation (23). Well-validated prenyltransferase inhibitors, such as FTI-277 (FTI), BMS-388891 (BMS), and GGTI-298 (GGTI), directly reduce levels of protein prenylation in P. falciparum (28, 44) (Fig. 1A). We tested whether inhibition of prenylphosphate synthesis or prenyltransferases influenced parasite growth following heat (40°C) or cold (25°C) stress. These temperatures were selected to emulate temperatures in which the parasite is exposed during febrile episodes (for heat stress) and during transmission to the mosquito vector (for cold stress). Parasites were pretreated with inhibitor 24 h prior to temperature shock. Parasite growth was evaluated by flow cytometry (Fig. S1) for 6 days postshock (Fig. 1B).

10.1128/mBio.00760-21.3 FIG S1 Gating strategy for infected RBCs. Uninfected erythrocytes were used as a control for gating purposes. (A) Total RBCs were gated based on the side scatter and forward scatter (SSC-A/FSC-W) profile in the dot plot. (B to G) Parasite-infected RBCs were gated based on forward scatter and fluorescein isothiocyanate (FSC-W/FITC-A). Gating was kept consistent between all treatment groups. Differences in number of parasite-infected RBCs are easily visible in dot plots. For example, heat-shocked FSM-treated parasites had fewer infected RBCs (E). Farnesol (F-OL) can rescue this temperature-sensitive growth (G). Download FIG S1, JPG file, 2.2 MB. Copyright © 2021 Mathews et al. 2021 Mathews et al. https://creativecommons.org/licenses/by/4.0/ This content is distributed under the terms of the Creative Commons Attribution 4.0 International license.

While untreated parasites readily recovered following brief heat or cold shock (Fig. 1C and D), we found that parasite survival under heat (Fig. 1C) or cold (Fig. 1D) stress was significantly attenuated upon nonlethal inhibition of IPP synthesis by FSM. FTI and BMS are chemically unrelated, well-validated protein farnesyltransferase inhibitors, while GGTI inhibits protein geranylgeranylation. Using these inhibitors, we found that chemical inhibition of protein farnesylation, but not geranylgeranylation, significantly impaired temperature stress recovery (Fig. 1E; Fig. S2).

10.1128/mBio.00760-21.4 FIG S2 Protein farnesylation, but not geranylgeranylation, is required for thermotolerance in malaria parasites. Parasites were treated with farnesyltransferase inhibitors (FTI [10 μM] and BMS [200 nM]) or geranylgeranyltransferase inhibitor GGTI (2μM) for 24 h prior to a 6-h heat (40°C) or cold (25°C) shock. (A to F) Inhibition of farnesylation (FTI or BMS treatment) significantly reduced growth after temperature stress. Growth in GGTI-treated parasites is unchanged after heat or cold shock. (A to F) *n* = 6; *, *P* ≤ 0.05; **, *P* ≤ 0.01; ***, *P* ≤ 0.001; ****, *P* ≤ 0.0001, 2-way ANOVA, *P* values adjusted for multiple comparisons using Sidak’s multiple-comparison test. Abbreviations: ctrl, control, hs, heat shock, cs, cold shock. Download FIG S2, JPG file, 0.7 MB. Copyright © 2021 Mathews et al. 2021 Mathews et al. https://creativecommons.org/licenses/by/4.0/ This content is distributed under the terms of the Creative Commons Attribution 4.0 International license.

**FIG 2:**
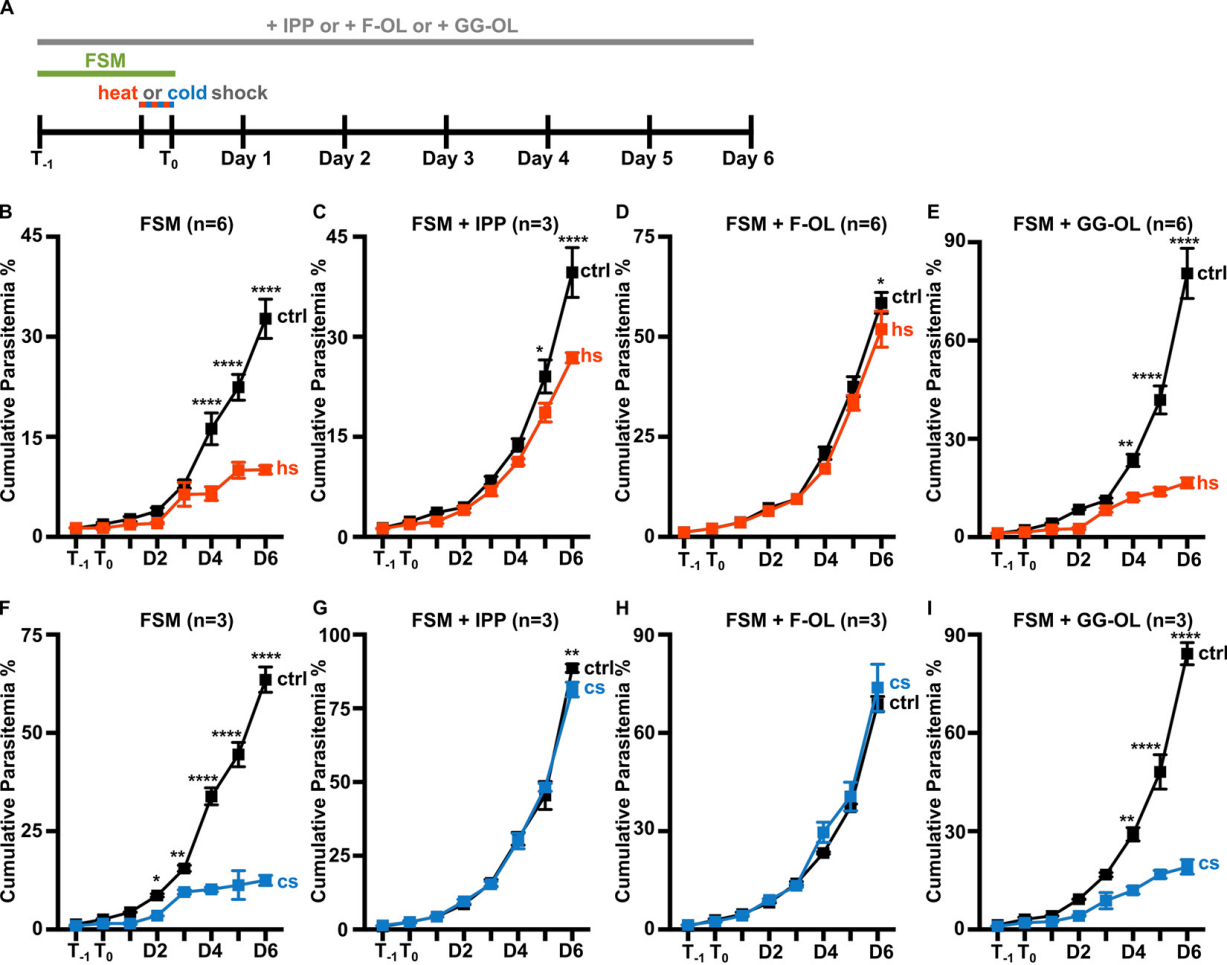
Supplementation with IPP and F-OL rescues growth in FSM-treated parasites after temperature stress. (A) Parasites were treated with FSM (5 μM) for 24 h prior to a 6-h heat (40°C) or cold (25°C) shock. Cultures were supplemented with isoprenoid products (IPP [250 μM], F-OL [10 μM], or GG-OL [10 μM]) for the entire length of experiment. (B) FSM-treated parasites are sensitive to heat shock. (C and D) Supplementation with IPP (C) or F-OL (D) rescues heat sensitivity. (E) GG-OL supplementation is unable to rescue growth after heat stress. (F to I) IPP or F-OL, but not GG-OL, supplementation is similarly able to rescue FSM-treated parasite growth after cold shock. (B to I) *n* = 3 to 6; *, *P* ≤ 0.05; **, *P* ≤ 0.01; ***, *P* ≤ 0.001; ****, *P* ≤ 0.0001. Two-way ANOVA, *P* values adjusted for multiple comparisons using Sidak’s multiple-comparison test. Abbreviations: ctrl, control, hs, heat shock, cs, cold shock.

Taking advantage of the fact that FSM inhibits production of all isoprenoid products downstream of IPP, we employed chemical supplementation in order to determine which isoprenoids are required for temperature stress survival in malaria parasites. We found that supplementation with IPP or farnesol (F-OL), a 15-carbon farnesyl alcohol, rescued FSM-treated parasites after both heat and cold stress (Fig. 2B to D and F to H). In contrast, supplementation with geranylgeraniol (GG-OL), a 20-carbon geranylgeranyl alcohol, did not rescue growth of FSM-treated parasites after temperature shock (Fig. 2E and I). Altogether, these data establish that loss of isoprenoid biosynthesis or protein farnesylation sensitizes malaria parasites to changes in temperature. Protein farnesylation is thus required for parasite survival following moderate, nonlethal temperature stress, in which heat shock proteins have a classic biological role. Only 4 farnesylated proteins have been identified in P. falciparum, 2 SNARE proteins (PF3D7_1324700, PF3D7_0910600), a PI3P binding protein (PF3D7_1460100), and the sole canonical HSP40 (PF3D7_1437900) (30, 31). Since HSP40 is predicted to modulate the activity of HSP70, a cellular chaperone required for heat survival (32), these data raise the possibility that farnesylation of HSP40 might have a vital role in parasite thermotolerance.

### HSP40 is resistant to disruption in P. falciparum.

We next examined the specific role of HSP40 in asexual parasite growth. HSP40 is one member of an expanded Hsp40 chaperone family in P. falciparum. While the majority of these Hsp40 family members do not have assigned biological functions, HSP40 is considered the canonical member of this family and directly interacts with HSP70 (PF3D7_0818900) (36). Based on forward genetic screening in P. falciparum, HSP40 is predicted to be essential during asexual blood-stage growth (45). To address the role of HSP40 in asexual development, we sought to disrupt the HSP40 locus directly. Using single-crossover homologous recombination (as previously used to validate the MEP pathway genes, Dxr and IspD) (20, 46), we successfully integrated a control plasmid into the HSP40 genetic locus (Fig. S3A and B). However, even after 7 months of continuous culture, integration of a disruption construct did not occur (Fig. S3A). Our data support an essential role for HSP40 in the parasite during blood-stage growth.

10.1128/mBio.00760-21.5 FIG S3 HSP40 locus is not amenable to disruption. Using single-crossover homologous recombination, we successfully integrated a control plasmid (pCAM-BSD HSP40^ctrl^) into the HSP40 (PF3D7_1437900) locus. Despite 7 months of continuous culture, integration of a disruption construct (pCAM-BSD HSP40^KO^) was not observed. (A) Genomic DNA isolated from blasticidin-resistant parasites transfected with pCAM-BSD HSP40^ctrl^ or pCAM-BSD HSP40^KO^ was subjected to PCR. Two sets of primers detect episomal plasmid in both cases (ctrl: 1,081 bp, 1,133 bp and KO: 902 bp, 982 bp). Integration events are detected by primers (∼1,800 bp). Integration of the control plasmid is present, while no integration of the KO plasmid is observed. Representative of three independent transfections. (B and C) Genomic DNA isolated from blasticidin-resistant parasites transfected with pCAM-BSD HSP40^ctrl^ or pCAM-BSD HSP40^KO^ was subjected to Southern blotting. (B) Plasmid and genomic DNA were digested with the restriction enzyme SmlI, transferred to membrane, and probed with a 719-bp HSP40 control fragment. The control plasmid (pCAM-BSD HSP40^ctrl^) was successfully integrated into the HSP40 locus in four independent transfections (1 to 4). Download FIG S3, JPG file, 0.7 MB. Copyright © 2021 Mathews et al. 2021 Mathews et al. https://creativecommons.org/licenses/by/4.0/ This content is distributed under the terms of the Creative Commons Attribution 4.0 International license.

### HSP40 can stimulate ATPase activity of its cochaperone HSP70 *in vitro*.

In contrast to the highly expanded HSP40 protein family, the P. falciparum genome encodes only 6 Hsp70-like proteins (34, 47). HSP70 (PF3D7_0818900), the major cytosolic Hsp70 in P. falciparum, possesses ATPase activity and is itself important for thermal tolerance in the parasite (32, 48–50). Select Hsp40 cochaperones interact with Hsp70 through a protein domain called a J domain (51–55). To determine whether HSP40 interacts with HSP70 to promote ATP hydrolysis, recombinant 6×His-HSP40 and 6×His-HSP70 were expressed and purified from Escherichia coli (Fig. S4A). We found that the addition of purified recombinant HSP40 stimulates the basal ATP hydrolytic activity of HSP70 (Fig. S4B and C). Increasing the amount of HSP70 in the reaction increases ATP turnover (Fig. S4D); however, the addition of HSP40 increases the ATPase activity of HSP70 nearly 3-fold (Fig. S4C). These data, along with previous observations by Botha et al. (36), confirm the functional interaction between HSP40 and HSP70.

10.1128/mBio.00760-21.6 FIG S4 ATPase activity of purified HSP70 and generation of polyclonal anti-HSP40 antisera that is specific for HSP40. (A) Recombinant HSP70 (73 kDa) and HSP40 (48 kDa) were purified from E. coli. Proteins are visualized on SDS gel with Coomassie blue stain. (B) The ATPase activities of 45 μg of HSP70 were measured every 12 s for 40 min. HSP70 had more ATPase activity in the presence of HSP40 (8.9 μg; green) than alone (blue). HSP40 (gray) and no protein (black) were used as controls, *n* = 4. (C) The slopes of the lines in panel B. (D) The rate of ATP turnover increases linearly with increased HSP70 concentration, *n* = 2. (E) Prebleed antisera immunoblot of recombinant protein, RBC lysate, and wild-type P. falciparum lysate. (F) Anti-HSP40 immunoblot of recombinant protein, RBC lysate, and wild-type P. falciparum lysate. A single band is observed at 48 kDa in parasite lysate corresponding to the expected size of HSP40. (E and F) Prebleed and anti-HSP40 were used at 1:5,000. (G) Spectral counts from Hsp40s identified by mass spectrometry after IP of parasite lysate with prebleed antisera or anti-HSP40. Fold change is the ratio of the average spectral counts between anti-HSP40 and prebleed. HSP40 is the only Hsp40 protein with a robust fold change and protein coverage. Download FIG S4, JPG file, 1.8 MB. Copyright © 2021 Mathews et al. 2021 Mathews et al. https://creativecommons.org/licenses/by/4.0/ This content is distributed under the terms of the Creative Commons Attribution 4.0 International license.

### Robust HSP40 membrane association requires IPP synthesis and protein farnesylation.

Using purified recombinant 6×His-HSP40 (Fig. S4A), polyclonal antisera were generated. Immunoblotting with anti-HSP40 antisera revealed a single band in parasite lysate, which is not present in uninfected red blood cells (RBCs) (Fig. S4E and F). Antisera specificity was confirmed by immunoprecipitation (IP) and mass spectrometry, demonstrating exclusive immunoprecipitation of HSP40 without cross-reactivity to other Hsp40 family members in P. falciparum (Fig. S4G).

**FIG 3:**
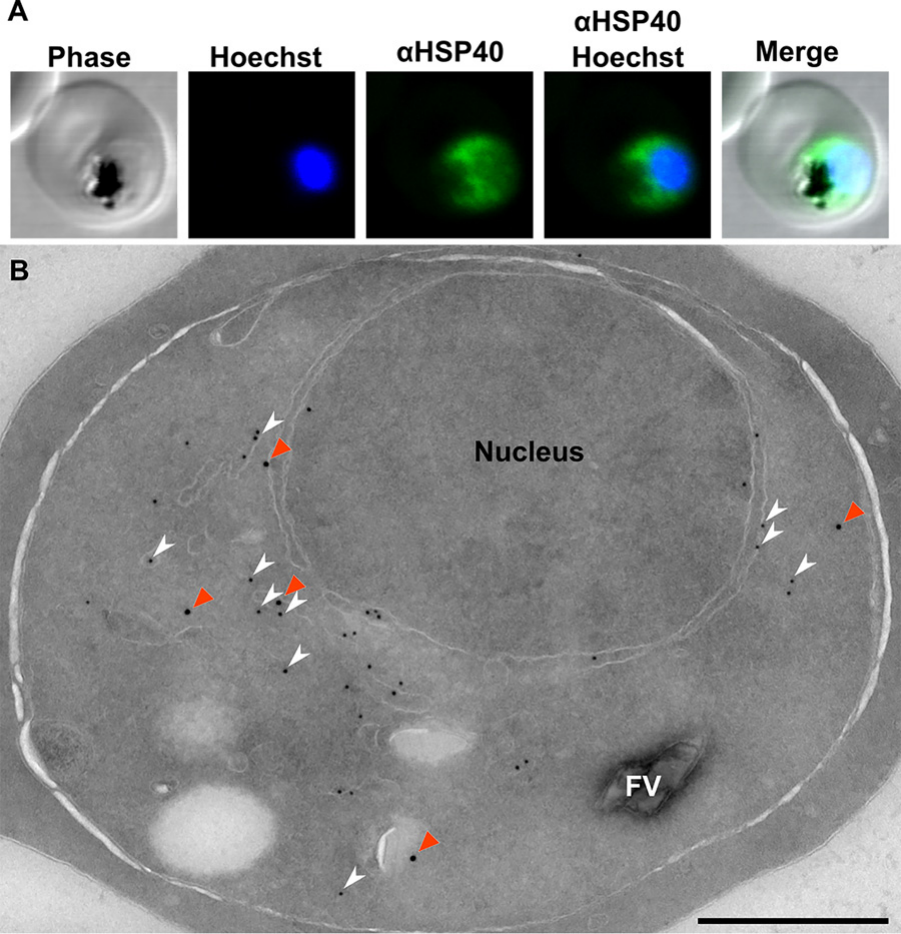
Localization of HSP40 in P. falciparum. (A) Immunofluorescence confocal microscopy of trophozoite, stained with anti-HSP40 (1:5,000) and Hoechst 33258 nuclear stain. HSP40 appears cytosolic. (B) Electron micrograph of immunolabeling: primary, rabbit anti-HSP40 (1:250), mouse anti-PDI (1:100); secondary, goat anti-rabbit IgG 18 nm colloidal gold, goat anti-mouse 12 nm. HSP40 (orange arrowheads) looks cytosolic in the parasites, with some apparent membrane association. A portion of HSP40 colocalizes with PDI, an established ER marker (white arrowheads). Scale, 500 nm.

Since heat shock proteins help mediate export of parasite proteins through the PTEX complex (56–58), we performed immunofluorescence localization of HSP40 in asexual P. falciparum. HSP40 localizes to the parasite cytosol in trophozoite-stage parasites and is not detected in the host cell (Fig. 3A). Immuno-electron microscopy (immuno-EM) was performed to further characterize the subcellular localization of HSP40. We found that HSP40 is distributed throughout the cytosol and excluded from the nucleus and host cell cytoplasm (Fig. 3B). Because farnesylation marks small GTPases for localization to the cytosolic face of the endoplasmic reticulum (ER) (59, 60), we predicted that some portion of HSP40 may be ER localized. In addition to anti-HSP40, parasites were labeled with anti-protein disulfide isomerase (anti-PDI), an established ER marker (61, 62). A subset of HSP40 (48.7% of all labeling) is localized alongside PDI in the ER (Fig. 3B). These data indicate that HSP40 is both cytosolic and ER localized.

**FIG 4:**
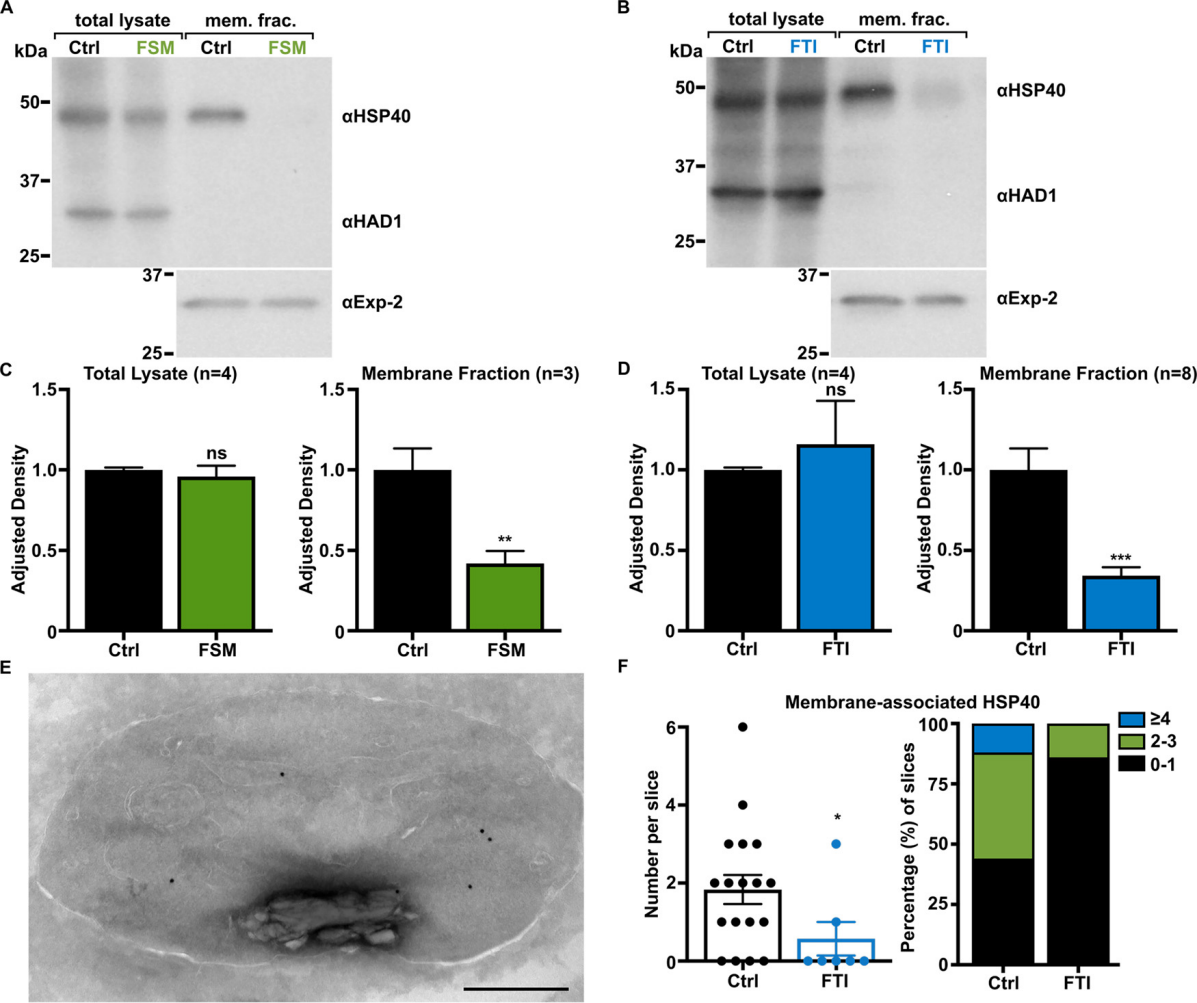
Inhibition of either IPP synthesis or protein farnesylation results in reduced membrane association of HSP40. (A and B) Representative anti-HSP40 immunoblots of control- and FSM (20 μM)-treated (A) or FTI (10 μM)-treated (B) P. falciparum total lysate and membrane fractions. (C and D) Quantification of several immunoblots adjusted to loading control. HSP40 is significantly reduced in the membrane fraction after inhibition of IPP synthesis (C) and inhibition of farnesylation (D). Anti-HAD1 and anti-Exp-2, loading controls for total lysate and membrane fractions, respectively. **, *P* ≤ 0.01; ***, *P* ≤ 0.001 unpaired *t* test with Welch’s correction. (E and F) HSP40 membrane association is reduced after FTI treatment. Apparent membrane-associated HSP40 (10 nm gold particles) is reduced after inhibition of farnesylation. The number of membrane-associated HSP40 per micrograph is quantified for control and treated parasites (F). A single control cohort was quantified. A decrease in the number of membrane-associated HSP40 particles is observed. *, *P* ≤ 0.05, unpaired *t* test with Welch’s correction. Scale, 500 nm.

HSP40 is found in the membrane fraction (Fig. 4A and B), following detergent fractionation of parasite lysate. While overall levels of HSP40 protein remain unchanged with inhibition of IPP synthesis or farnesylation, the percentage of membrane-associated HSP40 is significantly reduced (Fig. 4C and D). Treatment with farnesylation inhibitors (such as FTI or BMS), but not the geranylgeranylation inhibitor GGTI, reduces membrane-associated HSP40 (Fig. S5). Subcellular localization of HSP40 is changed after treatment with FTI (Fig. 4E). Quantification of immuno-EM micrographs confirms a reduced membrane association of HSP40 in inhibitor-treated cells (Fig. 4F). Overall, these observations provide evidence that a subset of HSP40 is membrane associated and that this membrane association requires farnesylation.

10.1128/mBio.00760-21.7 FIG S5 Farnesylation, not geranylgeranylation, mediates membrane association of HSP40. (A) Representative anti-HSP40 immunoblots of control-, BMS (200 nM)-, and GGTI (2μM)-treated P. falciparum total lysate and membrane fractions. (B) Quantification of several immunoblots adjusted with loading control. HSP40 is significantly reduced in the membrane fraction after inhibition of farnesylation (BMS) and not geranylgeranylation (GGTI). Anti-HAD1 and anti-Exp-2, loading controls for total lysate and membrane fractions, respectively. **, *P* ≤ 0.01, unpaired *t* test with Welch’s correction. Download FIG S5, JPG file, 0.7 MB. Copyright © 2021 Mathews et al. 2021 Mathews et al. https://creativecommons.org/licenses/by/4.0/ This content is distributed under the terms of the Creative Commons Attribution 4.0 International license.

### Palmitoylation contributes to HSP40 membrane association but not thermotolerance.

**FIG 5:**
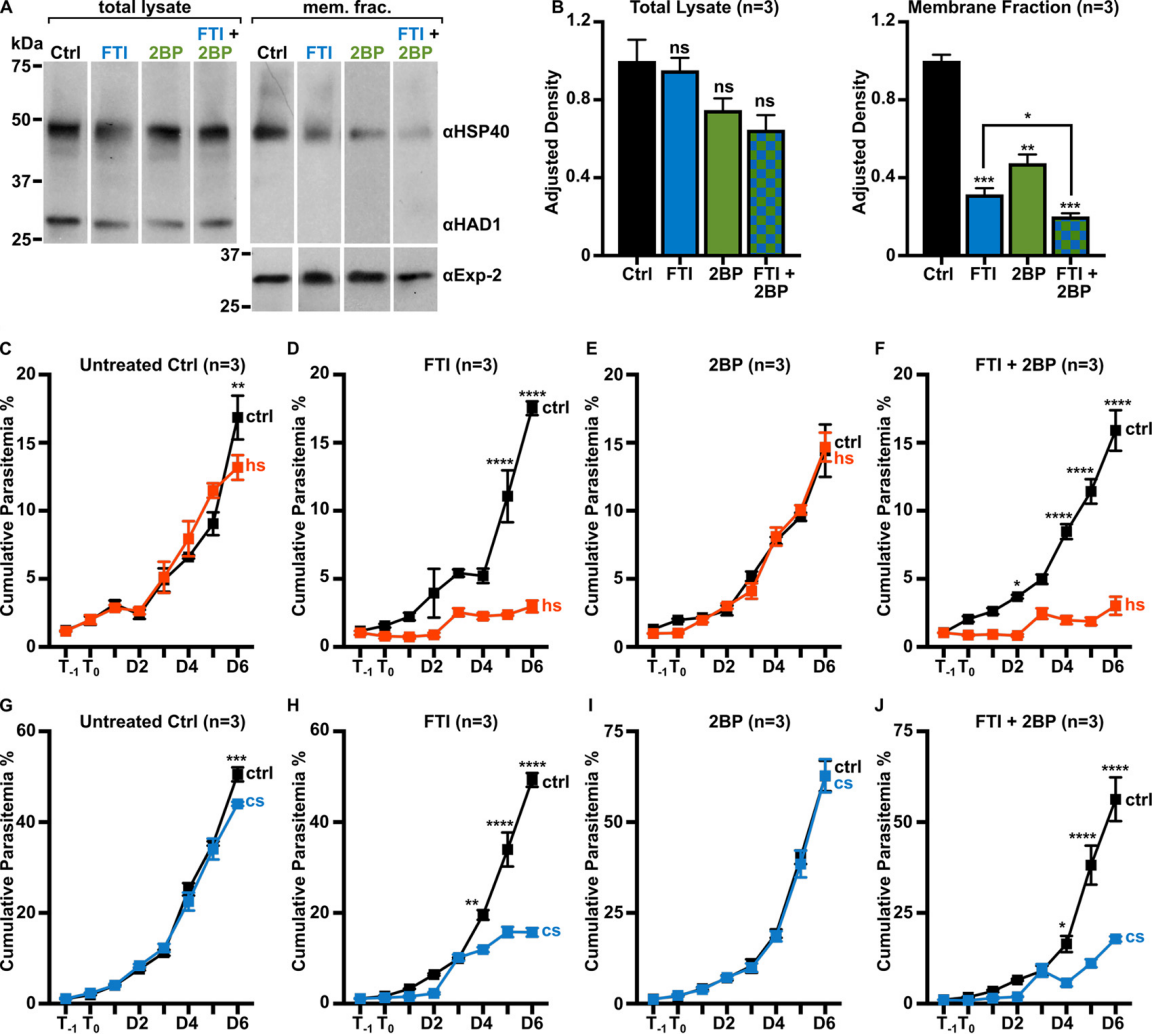
Both farnesylation and palmitoylation contribute to HSP40 membrane association, but only farnesylation is required for thermotolerance. (A) Representative anti-HSP40 immunoblots of control, FTI (10 μM), 2BP (100 μM), and combination of FTI- and 2BP-treated P. falciparum total lysate and membrane fractions. (B) Quantification of several immunoblots adjusted with loading control. The membrane-associated proportion of HSP40 is significantly reduced upon inhibition of farnesylation (FTI) or palmitoylation (2BP). Inhibition of both farnesylation and palmitoylation (FTI + 2BP) reduces HSP40 membrane association further than does single inhibitor treatment. Anti-HAD1 and anti-Exp-2, loading controls for total lysate and membrane fractions, respectively. *n* = 3; *, *P* ≤ 0.05; **, *P* ≤ 0.01; ***, *P* ≤ 0.001 unpaired *t* test with Welch’s correction. (C to J) Parasites were treated with FTI (10 μM), 2BP (100 μM), or both prior to heat (40°C) or cold (25°C) shock. FTI-treated parasite growth is significantly reduced after heat (D) and cold shock (H). Growth in 2BP-treated parasites is unchanged after heat or cold shock (E and I). Parasites treated with both FTI and 2BP were sensitive to temperature stress (F and J). *n* = 3; *, *P* ≤ 0.05; **, *P* ≤ 0.01; ***, *P* ≤ 0.001; ****, *P* ≤ 0.0001, 2-way ANOVA, *P* values adjusted for multiple comparisons using Sidak’s multiple-comparison test. Abbreviations: ctrl, control; hs, heat shock; cs, cold shock.

Farnesylation is not the only posttranslational modification that is expected to bring HSP40 to the membrane, as HSP40 is also palmitoylated (63). To evaluate the role of palmitoylation in the membrane association of HSP40 and parasite thermotolerance, we employed the palmitoylation inhibitor, 2-bromopalmitate (2BP) (64). While overall levels of HSP40 remained unchanged upon inhibition of palmitoylation, the proportion of membrane-associated HSP40 was significantly reduced (Fig. 5A and B). Combined treatment with 2BP and FTI (to inhibit both palmitoylation and farnesylation) further reduced the levels of membrane-associated HSP40 compared to either treatment alone (Fig. 5A and B). We tested whether inhibition of palmitoylation influenced parasite growth following heat (40°C) or cold (25°C) stress. Consistent with our previous observations, untreated parasites readily recovered after modest heat or cold shock (Fig. 5C and G), while inhibition of farnesylation significantly impaired recovery after temperature stress (Fig. 5D and H). In contrast, treatment with 2BP did not sensitize parasites to temperature stress, as growth was unchanged following heat and cold shock (Fig. 5E and I). Loss of protein palmitoylation was also not protective, as parasites treated with both FTI and 2BP remained sensitive to temperature shock (Fig. 5F and J). Therefore, while palmitoylation helped facilitate membrane association of HSP40, farnesylation, not palmitoylation, was required for parasite stress survival.

### Chemical inhibition of protein farnesylation alters the HSP40 interactome.

HSP40 is expected to interact with numerous cochaperones and client proteins. Protein prenylation is known to drive association with the ER and is likely to alter accessibility to client proteins (65). Therefore, we determined whether loss of prenylation alters the array of cellular client proteins that bind to HSP40. We used immunoprecipitation and mass spectrometry to identify HSP40-interacting proteins from parasites under normal prenylation conditions (wild-type controls) and when prenylation was chemically impaired either through inhibition of isoprenoid biosynthesis (with FSM) or through inhibition of farnesyl transferase activity (with FTI).

**FIG 6:**
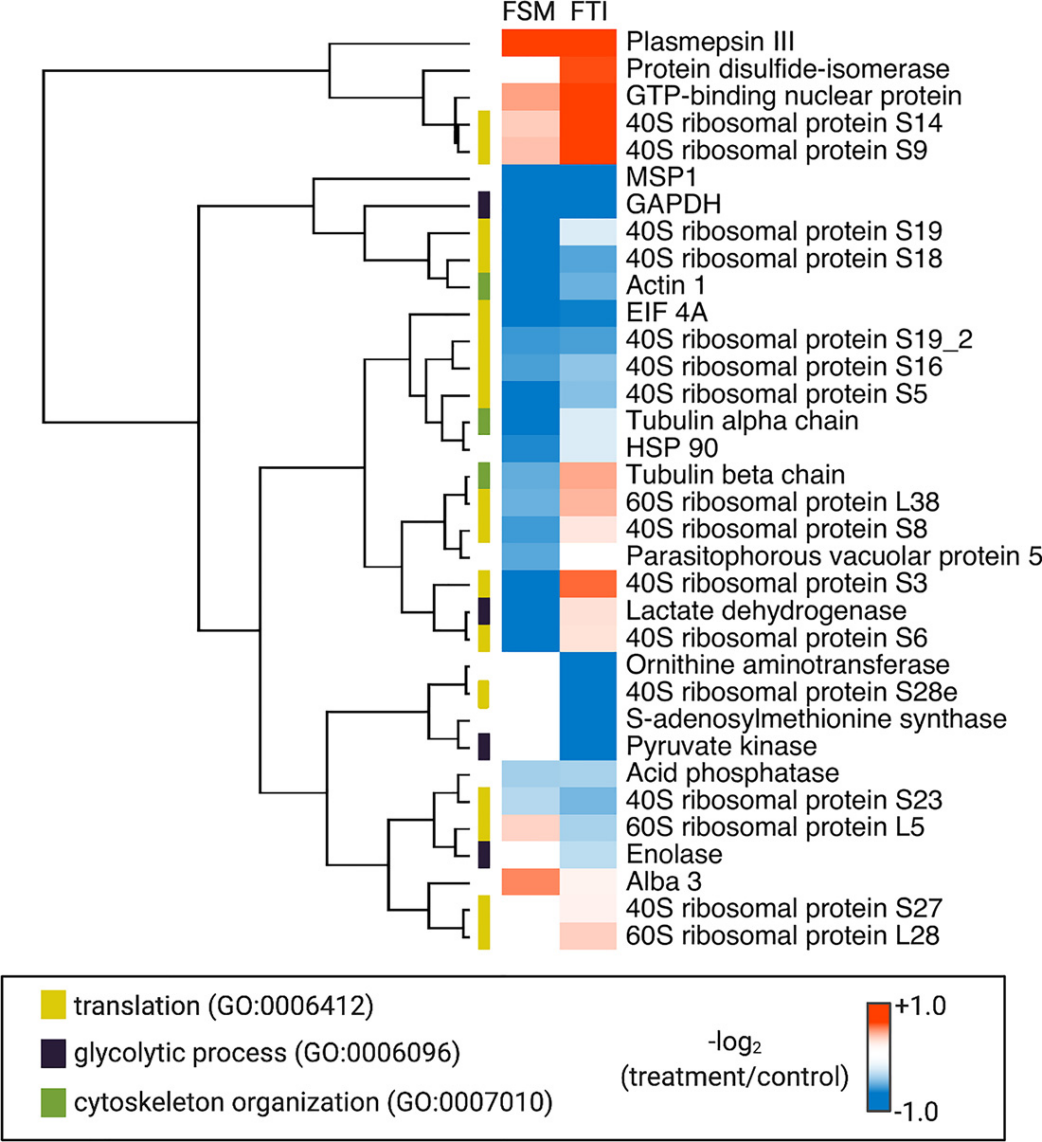
Both IPP synthesis and protein farnesylation influence HSP40 protein-protein interactions. Candidate protein interactors were determined by mass spectrometry after IP of parasite lysate with anti-HSP40. Results for FSM (5 μM)- and FTI (10 μM)-treated parasites are compared to untreated controls (*n* = 3). UniProt and GeneIDs are provided in Table S2. Heat map of normalized log_2_-transformed data was generated using NG-CHM Heat Map Builder. Gene Ontology (GO) annotations are indicated by colored bars.

We found that HSP40-interacting proteins mediated a number of essential biological functions in the parasite, including cytoskeleton organization, glycolysis, and translation (Table S1; Fig. 6; Table S2). When prenylation was reduced, either by reducing production of isoprenyl groups (with the isoprenoid biosynthesis inhibitor FSM) or by reducing transfer of prenyl groups (with the farnesyl transferase inhibitor FTI), the overall profile of client protein interactions was markedly altered. When prenylation is reduced, HSP40 had a reduced association with membranes and with the ER (Fig. 4). Loss of prenylation reduces association with a number of ribosomal proteins, consistent with reduced association with the rough ER. In addition, the interaction of HSP40 with the glycolytic enzyme GAPDH (glyceraldehyde-3-phosphate dehydrogenase) was reduced after treatment with either FSM or FTI (Fig. 6).

10.1128/mBio.00760-21.1 TABLE S1 Candidate HSP40 interacting proteins. Proteins that immunoprecipitated with anti-HSP40 (PF3D7_1437900) antisera in three independent experiments, but not with negative-control prebleed sera from the same animals, were identified by mass spectrometry. List is limited to those proteins that associated with Hsp40 in the absence of drug treatment. Download Table S1, DOCX file, 0.01 MB. Copyright © 2021 Mathews et al. 2021 Mathews et al. https://creativecommons.org/licenses/by/4.0/ This content is distributed under the terms of the Creative Commons Attribution 4.0 International license.

10.1128/mBio.00760-21.2 TABLE S2 Gene/protein IDs and annotations of candidate HSP40-interacting proteins. List includes all proteins whose association with Hsp40 was altered upon treatment with fosmidomycin or farnesyl transferase inhibition. Download Table S2, DOCX file, 0.01 MB. Copyright © 2021 Mathews et al. 2021 Mathews et al. https://creativecommons.org/licenses/by/4.0/ This content is distributed under the terms of the Creative Commons Attribution 4.0 International license.

### HSP40 farnesylation influences GAPDH localization but not its glycolytic function.

**FIG 7:**
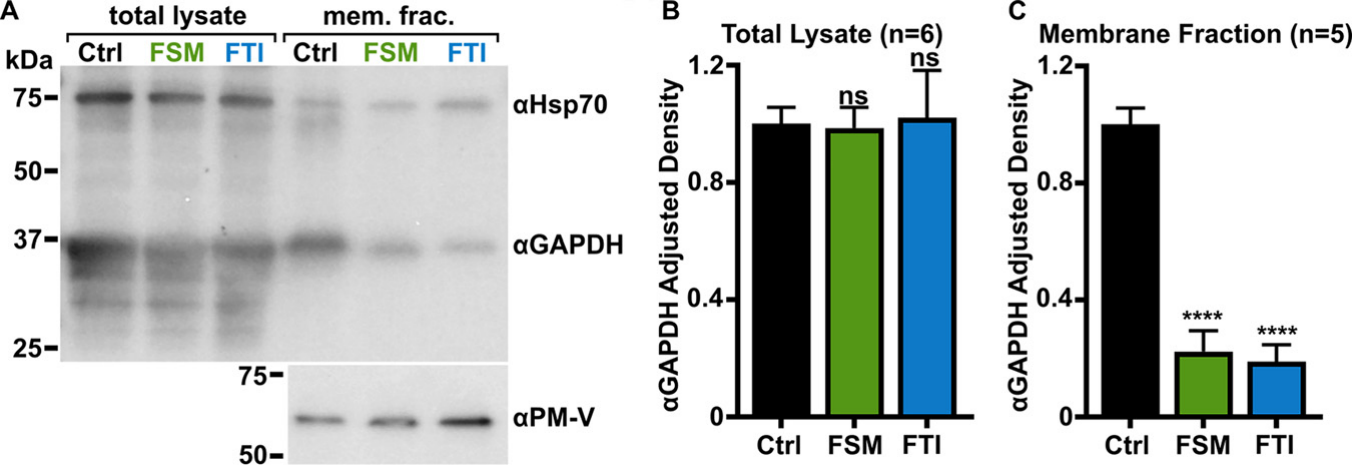
Localization of GAPDH, but not its glycolytic function, is IPP- or farnesylation-dependent. (A to C) Inhibition of IPP synthesis and farnesylation reduced membrane association of GAPDH. (A) Representative anti-GAPDH immunoblots of control-, FSM (20 μM)-, and FTI (10 μM)-treated P. falciparum. (B and C) Quantification of several immunoblots adjusted with loading control. Anti-Hsp70 (1:5,000) and anti-PM-V (1:500) were used as loading controls for total lysate and membrane fractions, respectively. *n* = 5 to 6; ****, *P* ≤ 0.0001, unpaired *t* test with Welch’s correction.

The farnesylation-dependent interaction between HSP40 and GAPDH (PF3D7_1462800) drew special attention because GAPDH is essential for the glycolytic breakdown of glucose to produce ATP in the parasite. Isoprenoid biosynthesis is immediately metabolically downstream of glycolysis, and therefore we further investigated the role of prenylation in mediating membrane association and function of GAPDH. Using purified recombinant 6×His-GAPDH, specific polyclonal antisera were generated (Fig. S6A and B). As observed for HSP40, we found that GAPDH was, in part, membrane associated, as has been previously observed (66, 67). We found that this association was dependent on both IPP synthesis and farnesylation (Fig. 7A to C).

10.1128/mBio.00760-21.8 FIG S6 Polyclonal anti-GAPDH antisera. (A) Prebleed antisera immunoblot of wild-type P. falciparum lysate, recombinant protein, and RBC lysate. (B) Anti-GAPDH immunoblot of wild-type P. falciparum lysate, recombinant protein, and RBC lysate. A single band is observed at 37 kDa in parasite lysate corresponding to the expected size of GAPDH. (A and B) Prebleed and anti-GAPDH were used at 1:1,000. Download FIG S6, JPG file, 0.5 MB. Copyright © 2021 Mathews et al. 2021 Mathews et al. https://creativecommons.org/licenses/by/4.0/ This content is distributed under the terms of the Creative Commons Attribution 4.0 International license.

**FIG 8:**
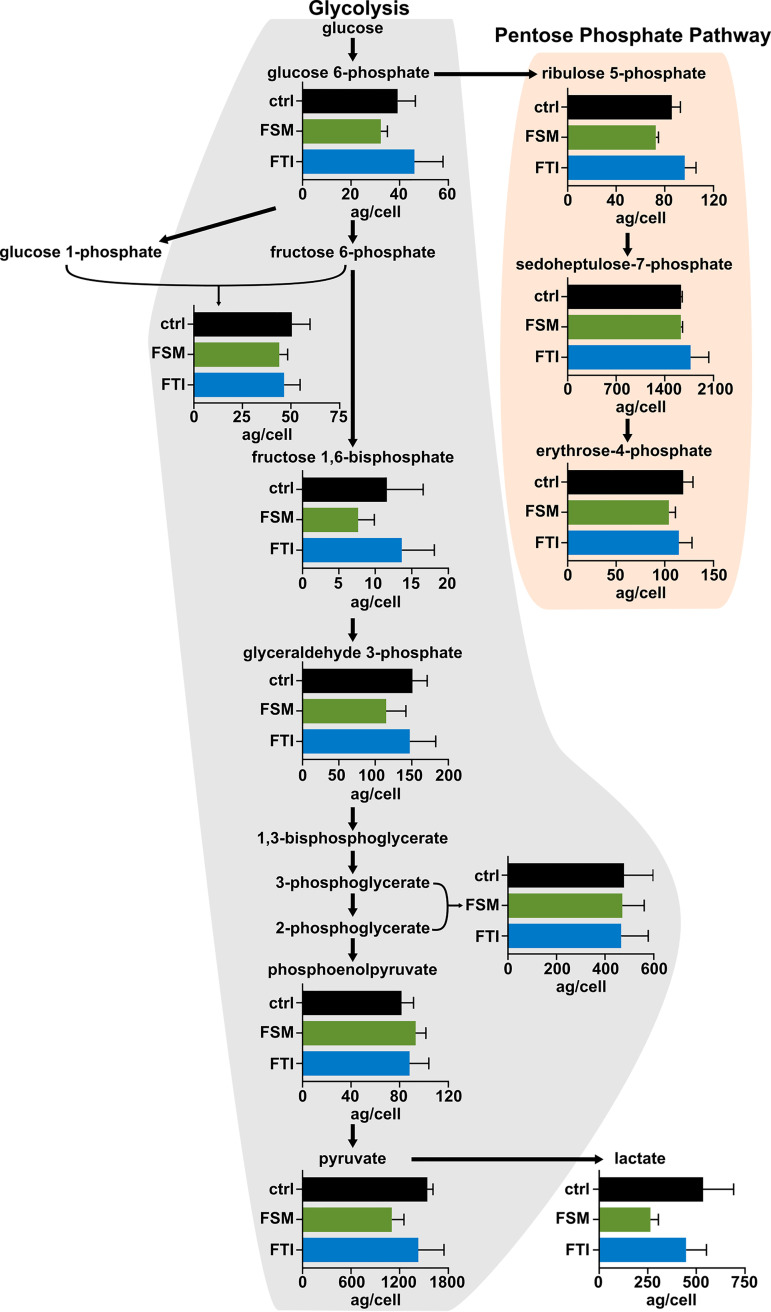
Glycolytic and pentose phosphate pathway metabolite levels remain constant under IPP- and farnesylation-deficient conditions. Levels of glycolytic and pentose phosphate pathway intermediates were measured by liquid chromatography with tandem mass spectrometry and normalized based on parasitemia of each individual sample to give concentration per cell. No significant changes are observed after treatment with FSM (5 μM) or FTI (10 μM). *n* = 3, unpaired *t* test with Welch’s correction.

To understand whether interrupting IPP synthesis and protein prenylation directly affects glycolytic function in the parasite, we quantified glycolytic intermediates in the presence and absence of IPP synthesis and protein prenylation. We found that the cellular levels of the products of glycolysis including pyruvate and lactate were unchanged after treatment with either FSM or FTI (Fig. 8). Substrate availability to the pentose phosphate pathway also did not change upon FSM or FTI treatment (Fig. 8). Our data indicate that, although the interaction between HSP40 and glycolytic enzymes is prenylation-dependent, farnesylation does not directly influence glycolytic function in the parasite.

## DISCUSSION

Temperature change is a critical environmental signal and an integral environmental stress of the P. falciparum life cycle. In addition, antimalarial activity of the first-line artemisinin-based therapies may be sensitive to heat or cold stress (6, 8). However, the mechanisms by which the parasite copes with thermal stress are not well understood, and the inherent host temperature fluctuations during clinical malaria may be exploited to improve malaria treatment. In this study, we reveal that survival during heat or cold shock in P. falciparum requires both *de novo* isoprenoid biosynthesis and the posttranslational 15-carbon isoprenyl modification called farnesylation. Chemical inhibitors that reduce protein farnesylation, by reducing either isoprenoid biosynthesis or farnesyltransferase activity, sensitize P. falciparum to otherwise nonlethal temperature stresses, even at otherwise subinhibitory doses. Our observations are supported by a parallel study by Zhang et al. (68), which also identified isoprenoid biosynthesis as critical for survival of P. falciparum at febrile temperatures, using a large-scale piggyBac transposon mutant phenotypic screen. Together, these observations suggest that isoprenoid and prenylation inhibitors might be particularly valuable in the setting of malarial fever, a nearly universal characteristic of symptomatic P. falciparum infection (69).

Housed within the apicoplast organelle, isoprenoid biosynthesis through the MEP pathway is necessary for intraerythrocytic development of P. falciparum (18–20). Isoprenyl modification of proteins is a key essential function of the MEP pathway, as prenylation itself is essential for asexual development (23–27). However, the pleiotropic downstream effects brought on by loss of protein prenylation have not been fully elucidated. Kennedy et al. recently proposed a mechanistic model of “delayed death,” a phenotype of parasite demise during the second erythrocytic cycle following drug treatment, exhibited by antimalarials that target apicoplast maintenance. In this model, disruption of Rab protein geranylgeranylation and interruption of subsequent cellular trafficking are responsible for the growth arrest caused by loss of IPP production or protein prenylation (70). Geranylgeranylated Rab GTPase family members comprise the majority of prenylated proteins in P. falciparum (30, 31). Rab GTPases are also believed to contribute to the structural integrity of the digestive food vacuole of the parasite (23), which is linked to thermal tolerance (32). However, our data suggest that the phenotype in malaria parasites caused by loss of isoprenoid biosynthesis or protein prenylation is more complicated and that interruption of protein farnesylation (even when geranylgeranylation is preserved) also plays an important role. Both geranylgeranylation of Rab GTPases and farnesylation, likely of HSP40, are key to the essential nature of prenylation in P. falciparum.

Our data also suggest the presence of several distinct cellular pools of HSP40 that have different posttranslational modifications, subcellular localizations, and client protein interactomes. Maximal membrane association of HSP40 requires posttranslational palmitoylation. Palmitoylation may guide HSP40 to a membrane subcompartment that is different from that of farnesyl-HSP40. Similar findings have been described for mammalian Ras proteins, which, as for HSP40, are both farnesylated and palmitoylated. For Ras proteins, both posttranslational modifications are needed for stable plasma membrane association (71). As palmitoylation is not required for parasite thermotolerance, palmitoyl-HSP40 appears to have other, yet-unidentified functions in malaria parasites.

**FIG 9:**
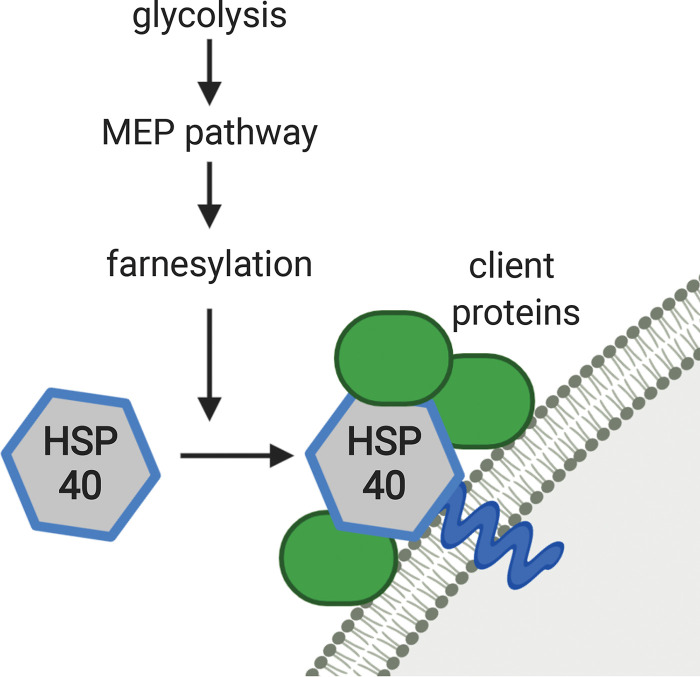
Isoprenoid biosynthesis and farnesylation affect membrane association and client protein assembly of HSP40.

While heat shock proteins are found across taxa, not all organisms possess prenylated Hsp40s (72). HSP40 orthologs in human, yeast, *Plasmodium* spp. (P. vivax, P. yoelii, P. chabaudi, and P. berghei), and plants (Arabidopsis thaliana and Atriplex nummularia) have been experimentally demonstrated to be prenylated or are predicted to be prenylated based on the presence of canonical C-terminal prenylation sequence motifs (37, 73–75). It is unclear how the function of Hsp40 orthologs might differ in organisms that lack prenylated Hsp40s. However, our data, in conjunction with studies in yeast and plants, provide compelling evidence that prenylation of Hsp40 has evolved for survival during environmental stress, including growth after temperature or drought stress (37–39, 74, 76, 77). HSP40 is one of only 4 farnesylated proteins in P. falciparum and the sole farnesylated heat shock protein (30, 31). We find that farnesylation controls membrane association of HSP40 on the endoplasmic reticulum, modulates access to its client proteins, and is necessary for survival during temperature stress (Fig. 9). Together, these observations suggest that prenylation of Hsp40 is a marker that distinguishes a distinct functional subclass of Hsp40s which appear to play similar cellular roles in animals, plants, and fungi and have essential functions in environmental stress responses that are conserved across the domains of life.

Our data suggest that compounds that target IPP synthesis or farnesylation may be more effective *in vivo* than *in vitro*, as parasites cycle through host temperature changes. As Hsp40 cochaperone family members are amenable to small molecule inhibition (78, 79), a combination therapy that targets both protein prenylation and heat shock proteins directly has the potential to be highly parasiticidal. In addition, Hsp40 function is likely important to survival under artemisinin treatment. Whole-genome sequencing of clinical parasite samples collected during the emergence of artemisinin resistance in Thailand revealed a nonsynonymous mutation in Hsp40 that may provide a suitable background for artemisinin resistance mutations (80). A recent study indicates that the P. falciparum transcriptional response to heat stress and artemisinin are highly correlated (68). Targeting apicoplast function, prenylation, or heat shock also hold promise as adjunctive therapies to reverse artemisinin susceptibility.

## MATERIALS AND METHODS

### Materials.

All buffer components, salts, and enzyme substrates were purchased from Millipore Sigma (Burlington, MA), unless otherwise indicated.

### Statistical analysis.

We plotted all data and performed all statistical analyses in GraphPad Prism software (version 8). All data are expressed as the mean ± standard error of the mean (SEM). For statistical analysis, we used 2-way analysis of variance (ANOVA), *t* test with Welch’s correction, and multiple unpaired *t* test to compare results. To understand the interaction between treatment and temperature stress, we utilized a 2-way ANOVA and then adjusted *P* values for multiple comparisons using Sidak’s multiple-comparison test. For direct comparisons between control and treatment groups, we employed *t* test with Welch’s correction because standard deviations were not the same between groups. Mass spectrometry data were analyzed using multiple *t* tests to efficiently compare results across conditions for each protein.

### Drug inhibitors and isoprenoids.

Fosmidomycin (50 mM; Millipore Sigma), FTI-277 (5 mM; Tocris Bioscience, Bristol, UK), and IPP (30 mM; Echelon Biosciences, Salt Lake City, UT) were each dissolved in water at concentrations indicated. GGTI-298 (20 mM; Cayman Chemical, Ann Arbor, MI), BMS-388891 (20 mM; kindly provided by Wesley Van Voorhis, University of Washington), 2-bromopalmitate (100 mM; Millipore Sigma), farnesol (50 mM; Millipore Sigma), and geranylgeraniol (50 mM; Echelon Biosciences) were each dissolved in 100% dimethyl sulfoxide (DMSO) at concentrations indicated.

### Parasite strains and culture.

Unless otherwise indicated, parasites were maintained at 37°C in 5% O_2_–5% CO_2_–90% N_2_ in a 2% suspension of human erythrocytes in RPMI medium modified with 27 mM NaHCO_3_, 11 mM glucose, 5 mM HEPES, 0.01 mM thymidine, 1 mM sodium pyruvate, 0.37 mM hypoxanthine, 10 μg/ml gentamicin, and 5 g/liter Albumax (Thermo Fisher Scientific, Waltham, MA). All experiments were conducted in wild-type strain 3D7 (MRA-102) obtained through the MR4 as part of the BEI Resources Repository, NIAID, NIH.

### Heat and cold shock P. falciparum growth assays.

Asynchronous cultures were diluted to 1% parasitemia. Cultures were treated with indicated drugs 24 h prior to a 6-h heat shock (40°C) or cold shock (25°C). Media (no drug) were exchanged post temperature shock. Cultures were split 1:6 after the collection of the day 2 sample. IPP, F-OL, and GG-OL were supplemented in fresh media every second day. Samples were taken at indicated time points and fixed in phosphate-buffered saline (PBS)–4% paraformaldehyde. Cells were stained with 0.01 mg/ml acridine orange, and parasitemia was determined on a BD Biosciences LSRII flow cytometer (Thermo Fisher Scientific). All data represent means of results from ≥3 independent experiments using biological replicates.

### pCAM-BSD HSP40 plasmid construction.

Functional genetic validation of HSP40 (PF3D7_1437900) was performed as described previously for PfDXR (20) and PfIspD (46). pCAM-BSD-HSP40^KO^ and pCAM-BSD-HSP40^ctrl^ vectors were derived from pCAM-BSD (gift from David Fidock, Columbia University), which includes a blasticidin-resistance cassette under transcriptional control by the P. falciparum D10 calmodulin 5′ untranslated region (UTR) and the 3′ UTR of HRP2 from P. berghei. To construct pCAM-BSD-HSP40^KO^, the coding sequence for a segment of HSP40 near the N terminus (bp 29 to 878) was inserted directly into 5′ of the P. berghei dihydrofolate reductase (DHFR)-thymidylate synthase 3′ UTR. This insert was constructed by PCR using primers (A) 5′-CCCGGGACTCTATGGGTGGTCAACAAG-3′ and (B) 5′-CTCGAGTCTCATGTAGGTCTTGGTTCC-3′ and restriction cloned using *Xma*I and *Xho*I sites. pCAM-BSD-HSP40^ctrl^ contained the coding sequence for the C-terminal end of HSP40 (bp 963 to 1619) and 237 bp of the 3′ UTR, generated using (C) 5′-CCCGGGGAGGAACCAAGACCTACATGAG-3′ and (D) 5′-CTCGAGCATTTCACAGACACACACACAC-3′ as primers. This sequence was inserted at the same site, 5′ of the P. berghei DHFR-thymidylate synthase 3′ UTR. All constructs were verified by Sanger sequencing.

### Parasite transfections.

Transfections were performed as described previously (46). Briefly, 150 μg of plasmid DNA was precipitated and resuspended in Cytomix (25 mM HEPES [pH 7.6], 120 mM KCl, 0.15 mM CaCl_2_, 2 mM EGTA, 5 mM MgCl_2_, 10 mM K_2_HPO_4_). A ring-stage P. falciparum culture was washed with Cytomix and resuspended in the DNA/Cytomix solution. Cells were electroporated using a Bio-Rad Gene Pulser II electroporator at 950 μF and 0.31 kV. Electroporated cells were washed with media and returned to normal culture conditions. Parasites expressing the construct were selected by continuous treatment with 2 μg/ml blasticidin S HCl (Thermo Fisher Scientific). Transfectants were cloned by limiting dilution, and diagnostic PCRs were performed using genomic DNA from resultant transfectants using primer sets specific for episomal plasmids or genome integrants. Primer A and D sequences are as follows: (X) 5′-TAAGAACATATTTATTAAACTGCAG-3′; (Y) 5′-GAAAAACGAACATTAAGCTGCCATA-3′.

### Southern blotting.

Southern blotting was used to assay the integration of the pCAM-BSD-HSP40^ctrl^ plasmid. To assay the integration of pCAM-BSD-PfHsp40^ctrl^, genomic DNA was harvested from wild-type 3D7 P. falciparum and from the continuously cultured pCAM-BSD-HSP40^ctrl^ transfectants 1, 2, 3, and 4 from Fig. S3. These genomic DNA samples, along with pCAM-BSD-HSP40^ctrl^ plasmid, were digested with SmlI (New England Biolabs). The control probe was prepared from PCR product generated using primers (HSP40_Ctrl_F) 5′-GAGGAACCAAGACCTACATGAG-3′ and (HSP40_Ctrl_R) 5′-ATGATCTTCATCGTCGTATGC-3′ (HSP40 bp 856 to 1,574) prior to Southern blotting.

### Generation of recombinant HSP40, HSP70, and GAPDH.

An E. coli codon optimized HSP40 was produced (Genewiz, South Plainfield, NJ) and inserted via ligation-independent cloning into the isopropyl-β-d-1-thiogalactopyranoside (IPTG) inducible BG1861 expression vector. This created an N-terminal 6×His tag fusion protein used for nickel purification. The expression plasmid was transformed into One Shot BL21(DE3)pLysS E. coli cells (Thermo Fisher Scientific). Overnight, starter cultures were diluted 1:1,000 and grown to an optical density (OD) of ∼0.6 where 1 mM IPTG was added for 16 h at 16°C. Cells were spun and stored at −80°C. Recombinant HSP70 (PF3D7_0818900) was expressed using the same conditions. In a similar manner, an E. coli codon optimized GAPDH (PF3D7_1462800) was produced with a few minor experimental differences. The GAPDH expression plasmid was transformed into One Shot BL21(DE3) E. coli cells (Thermo Fisher Scientific). One mM IPTG was added for 2 h at 37°C.

Expressed proteins were purified from cells using a sonication lysis buffer containing 1 mg/ml lysozyme, 20 mM imidazole, 1 mM dithiothreitol, 1 mM MgCl_2_, 10 mM Tris-HCl (pH 7.5), 30 U benzonase, 1 mM phenylmethylsulfonyl fluoride (PMSF), and cOmplete EDTA-free protease inhibitor tablets (Roche, Basel, Switzerland). Lysates were clarified using centrifugation, and proteins were purified via nickel agarose beads (Gold Biotechnology, Olivette, MO), eluted with 300 mM imidazole, 20 mM Tris-HCl (pH 7.5), and 150 mM NaCl. Eluted proteins were further purified via size exclusion chromatography using a HiLoad 16/60 Superdex 200 gel filtration column (GE Healthcare, Chicago, IL) using an AKTAExplorer 100 fast protein liquid chromatography (FPLC) system (GE Healthcare). Fast protein liquid chromatography buffer contained 100 mM Tris-HCl (pH 7.5), 1 mM MgCl_2_, 1 mM dithiothreitol (DTT), and 10% wt/vol glycerol. HSP70 and GAPDH fractions containing purified protein were individually pooled, concentrated to ∼2 mg/ml as determined via Pierce BCA protein assay kit (Thermo Fisher Scientific), and stored by adding 50% glycerol for storage at −20°C. HSP40 fractions containing purified protein were pooled and further purified via anion exchange using a Mono Q anion exchange chromatography column (GE Healthcare) using an AKTAExplorer 100 FPLC (GE Healthcare). Anion exchange buffer contained 100 mM Tris-HCl (pH 8.0), 1 mM MgCl_2_, and 100 mM NaCl. Purified fractions were concentrated to ∼2 mg/ml as determined via Pierce BCA protein assay kit (Thermo Fisher Scientific), glycerol was added to reach a concentration of 10% (wt/vol), and protein solutions were immediately flash frozen and stored at −80°C.

### HSP70 ATPase activity assays.

Hydrolysis of ATP by HSP70 was measured using an EnzChek phosphate assay kit (Thermo Fisher Scientific). All reaction mixtures contained 50 mM ATP. HSP70 was added to the reaction ranging from 9 to 45 μg. HSP40 was used in reactions at 8.9 μg. Absorbance was measured every 12 s for 40 min. Slopes were calculated by using the nonlinear regression analysis tool in Prism (GraphPad Software). All data represent means of results from ≥3 independent experiments using biological replicates and performed with technical replicates.

### HSP40 and GAPDH antiserum generation.

HSP40 and GAPDH rabbit polyclonal antisera was generated by Cocalico Biologicals (Reamstown, PA) using their standard protocol. Purified 6×His-HSP40 or 6×His-GAPDH was used as the antigen and TiterMax was used as an adjuvant. Antiserum specificity was confirmed by immunoblotting of parasite lysate, RBCs, and purified protein. Further confirmation of anti-HSP40 specificity was conducted by performing immunoprecipitation (IP) analysis (discussed in “Immunofluorescence and immuno-EM,” below).

### Immunofluorescence and immuno-EM.

For immunofluorescence labeling, infected RBCs at ∼8% parasitemia were fixed with 4% paraformaldehyde diluted in PBS. Cultures were treated with indicated drugs 24 h prior to collection. Fixed cells were washed with 50 mM ammonium chloride, permeabilized by treatment with 0.075% NP-40 in PBS, and blocked using 2% bovine serum albumin in PBS. Cells were incubated with 1:5,000 rabbit polyclonal anti-HSP40 (described in “HSP40 and GAPDH antiserum generation,” above). Hoechst 33258 (Thermo Fisher Scientific) was used as a nuclear counterstain. Dilutions of 1:1,000 of Alexa Fluor 488 goat anti-rabbit IgG (Thermo Fisher Scientific, A11008) were used as a secondary antibody. Images were obtained on an Olympus Fluoview FV1000 confocal microscope. For all immunofluorescence, minimal adjustments in brightness and contrast were applied equally to all images.

For immuno-EM, parasites were cultured at 2% hematocrit until they reached ∼6 to 8% parasitemia. Cultures were treated with indicated drugs 24 h prior to collection. Parasites were magnetically sorted from uninfected RBCs and ring-stage parasites via MACS LD separation columns (Miltenyi Biotech, Bergisch Gladbach, Germany). Parasites were collected by centrifugation and fixed for 1 h on ice in 4% paraformaldehyde in 100 mM PIPES [piperazine-*N*,*N*′-bis(2-ethanesulfonic acid)]/0.5 mM MgCl_2_ (pH 7.2). Samples were then embedded in 10% gelatin and infiltrated overnight with 2.3 M sucrose/20% polyvinylpyrrolidone in PIPES/MgCl_2_ at 4°C. Samples were frozen in liquid nitrogen and then sectioned with a Leica Ultracut UCT7 cryo-ultramicrotome (Leica Microsystems, Wetzlar, Germany). Fifty-nm sections were blocked with 5% fetal bovine serum/5% normal goat serum for 30 min and subsequently incubated with primary antibody for 1 h at room temperature. Primary antibodies used include anti-HSP40 (1:250) and anti-PDI (1D3) mouse 1:100 (ADI-SPA-891-D; Enzo Life Sciences, Farmingdale, NY). Secondary antibodies were added at 1:30 for 1 h at room temperature. Secondary antibodies included 12 nm Colloidal Gold AffiniPure goat anti-mouse IgG (H + L) (115-205-146; Jackson ImmunoResearch, West Grove, PA) and 18 nm Colloidal Gold AffiniPure goat anti-rabbit IgG (H + L) (111-215-144; Jackson ImmunoResearch). Sections were then stained with 0.3% uranyl acetate/2% methyl cellulose and viewed on a JEOL 1200 EX transmission electron microscope (JEOL USA Inc., Peabody, MA) equipped with an AMT 8 megapixel digital camera and AMT Image Capture Engine V602 software (Advanced Microscopy Techniques, Woburn, MA). All labeling experiments were conducted in parallel with controls omitting the primary antibody. Quantification of membrane-associated HSP40 was performed in micrographs where both a nucleus and food vacuole were present. Images were blinded and scored for total number labeled HSP40 and membrane-associated HSP40 (defined as being on or directly touching a well-visualized membrane).

### Membrane fraction preparation and immunoblotting.

Cultures were treated with indicated drugs 24 h prior to collection. Asynchronous parasites were released from RBCs with 0.1% saponin, washed in cold PBS, and resuspended in 100 to 300 μl deionized (DI) water with 1 mM PMSF and cOmplete EDTA-free protease inhibitor tablet (Roche). Resuspended pellets were freeze-thawed three times with liquid nitrogen/37°C water bath. A total lysate sample was taken at this point in the protocol. The membranes were pelleted (14,000 RPM, 30 min, 4°C), and the supernatant was collected as the soluble fraction. Pellets were washed once with ice-cold PBS and pelleted (as before) before pellets were resuspended in 100 to 300 μl (depending on sample amount) radioimmune precipitation assay (RIPA) buffer (Cell Signaling Technology, Danvers, MA) containing 1% CHAPS {3-[(3-cholamidopropyl)-dimethylammonio]-1-propanesulfonate} and 1% ASB-14 (amidosulfobetaine-14). Samples were sonicated three times with a microtip and incubated at 42°C with shaking at 800 RPM for 45 min. The samples were then centrifuged (14,000 RPM, 30 min, 4°C), and the resulting supernatant was collected as the membrane fraction. A sample buffer of 4× SDS was added, and samples were boiled for 10 min and loaded on 4 to 20% mini-PROTEAN TGX gradient gels (Bio-Rad Laboratories, Hercules, CA).

For immunoblotting, proteins were transferred onto PVDF using wet transfer with 20% methanol. Blots were blocked either 1 h at 25°C or overnight at 4°C with 2% bovine serum albumin–0.1% Tween 20–PBS. Primary antibodies were used at the following dilutions: 1:5,000 rabbit anti-HSP40, 1:5,000 rabbit anti-GAPDH, 1:10,000 rabbit anti-HAD1 (81), 1:5,000 rabbit anti-Hsp70 (AS08 371; Agrisera Antibodies, Vännäs, Sweden), 1:500 mouse anti-PM-V (82), and 1:5,000 mouse anti-EXP-2 clone 7.7 (83). For all blots, 1:20,000 horseradish peroxidase (HRP)-conjugated goat anti-rabbit IgG antibody (Thermo Fisher Scientific, 65-6120) or 1:5,000 HRP-conjugated goat anti-mouse IgG antibody (Thermo Fisher Scientific, G-21040) was used as the secondary antibody. Anti-HAD1 or anti-Hsp70 and anti-PM-V or anti-Exp-2 were used as loading controls from total lysate and membrane fractions, respectively. All blot images are representative of results of a minimum of 3 independent experiments using biological replicates.

### Immunoprecipitation of HSP40 from parasite lysate and protein mass spectrometry.

Preinoculated rabbit antisera and anti-HSP40 were coupled to magnetic beads using the Dynabeads antibody coupling kit as per the manufacturer’s protocol (Thermo Fisher Scientific, 14311D). Asynchronous parasite pellets (100 ml at 4% hematocrit and roughly 10% parasitemia) were harvested from RBCs using 0.1% saponin. Cultures were treated with indicated drugs 24 h prior to collection. Isolated parasite pellets were lysed in 300 μl of buffer containing 25 mM Tris-HCl (pH 7.5), 100 mM NaCl, 5 mM EDTA, 0.5% Triton-X 100, and 1 cOmplete mini EDTA-free protease inhibitor tablet (Roche) (per 10 mL of buffer). Resuspended pellets were homogenized using a LabGEN homogenizer (Cole-Parmer, Vernon Hills, IL) in 3 rounds of 30-s intervals with 60 s of rest on ice. Lysate was centrifuged at 14,000 RPM for 10 min at 4°C. The resulting soluble lysate was diluted by adding 450 μl of binding buffer containing 25 mM Tris-HCl (pH 7.5) and 150 mM NaCl. Diluted lysate was added to antisera-coupled beads that were previously washed three times with the same binding buffer. Lysate and beads were rotated for 2 h at 4°C. Beads were then washed three times with wash buffer (25 mM Tris-HCl [pH 7.5] and 500 mM NaCl) and flowthrough was discarded. Immunoprecipitated proteins were eluted using elution buffer with 200 mM glycine (pH 2.5) for 30 s, and eluted sample was neutralized with 1 M Tris-HCl (pH 7.5). Samples were then flash frozen and stored at −80° for immunoprecipitate identification via protein mass spectrometry.

Immunoprecipitates were identified via protein mass spectrometry by the Proteomics and Mass Spectrometry Core at the Donald Danforth Plant Science Center (St. Louis, MO) and analyzed by the Proteomics Core facility of the Children’s Hospital of Philadelphia. Stored samples were submitted in solution for protein mass spectrometry. Protein and peptide identification/quantification was performed with MaxQuant (1.6.14.0) using a Plasmodium falciparum reference database from UniProt (UP000001450). Carbamidomethyl of Cys was defined as a fixed modification. Oxidation of Met and acetylation of protein N-terminal were set as variable modifications. Trypsin/P was selected as the digestion enzyme, and a maximum of 3 labeled amino acids and 2 missed cleavages per peptide were allowed. Fragment ion tolerance was set to 0.5 Da. The tandem mass spectrometry (MS/MS) tolerance was set at 20 ppm. The minimum peptide length was set at 7 amino acids. The false discovery rate for peptides and proteins was set at 1%. The rest of the parameters were kept as default.

Perseus (1.6.14.0) was used for proteomics data processing and statistical analysis. The MaxLFQ intensity values were used to analyze the whole-cell proteome data. Protein groups containing matches to decoy database or contaminants were discarded. The data were log_2_ transformed and normalized by subtracting the median for each sample. Proteins with fewer than two values in each group were filtered out. Nonspecific binding proteins were removed by filtering proteins that were enriched in pull-downs from prebleed sera. A heat map of normalized log_2_-transformed data was generated using NG-CHM Heat Map Builder (84). PANTHER 16.0 was used to annotate Gene Ontology (GO) terms for each gene (85, 86)

### Metabolite profiling.

A total of 60 ml of sorbitol-synchronized early trophozoites cultured at 4% hematocrit until it reached ∼7 to 11% parasitemia was isolated using 0.1% saponin, washed with ice-cold PBS, and frozen at −80°C. Glycolysis and pentose phosphate pathway intermediates were extracted via the addition of glass beads (212 to 300 μm) and 600 μl chilled H_2_O–chloroform–methanol (3:5:12 vol/vol) spiked with PIPES [piperazine-*N*,*N*′-bis(2-ethanesulfonic acid)] as the internal standard. The cells were disrupted with the TissueLyser II instrument (Qiagen, Hilden, Germany) using a microcentrifuge tubes adaptor set prechilled for 2 min at 20 Hz. The samples were then centrifuged at 16,000 × *g* at 4°C, the supernatants were collected, and the pellet extraction was repeated once more. The supernatants were pooled, and 300 μl of chloroform and 450 μl of chilled water were added to the supernatants. The tubes were vortexed and centrifuged. The upper layer was transferred to a new tube and dried using a speed-vac. The pellets were redissolved in 100 μl of 50% acetonitrile.

For liquid chromatography (LC) separation of the glycolysis/pentose phosphate pathway intermediates, an InfinityLab Poroshell 120 HILIC (2.7 μm, 150 by 2.1 mm, Agilent) was used flowing at 0.5 ml/min. The gradient of the mobile phases A (20 mM ammonium acetate [pH 9.8], 5% acetonitrile [ACN]) and B (100% acetonitrile) was as follows: 85% B for 1 min, to 40% B in 9 min, hold at 40% B for 2 min, then back to 85% B in 0.5 min. The liquid chromatography (LC) system was interfaced with a Sciex QTRAP 6500+ mass spectrometer equipped with a TurboIonSpray (TIS) electrospray ion source. Analyst software (version 1.6.3) was used to control sample acquisition and data analysis. The QTRAP 6500+ mass spectrometer was tuned and calibrated according to the manufacturer’s recommendations. Metabolites were detected using MRM (multiple reaction monitoring) transitions that were previously optimized using standards. The instrument was set up to acquire in negative mode. For quantification, an external standard curve was prepared using a series of standard samples containing different concentrations of metabolites and fixed concentration of the internal standard. The limits of detection for glycolysis and pentose phosphate pathway intermediates were as follows: glucose 6-phosphate and glucose 1-phosphate/fructose 6-phosphate, 0.5 μM; glyceraldehyde 3-phosphate and ribulose 5-phosphate, 1 μM; erythrose-4-phosphate, 1.5 μM; pyruvate, 2/3-phosphoglycerate, phosphoenolpyruvate, and sedoheptulose-7-phosphate, 2 μM; fructose 1,6-bisphosphate, 3.9 μM. Resulting metabolite levels were normalized to parasitemia levels for each individual sample and reported as attogram per cell (ag/cell). Levels were averaged between three biological replicates and compared between control, FSM (5 μM for 24 h), and FTI (10 μM for 24 h).
