# Supplementary figures and images for: Protein Prenylation and Hsp40 in Thermotolerance of Plasmodium falciparum Malaria Parasites
Source: mBio. 2021 Jun 29;12(3):e00760-21. doi: 10.1128/mBio.00760-21 (PMC8262983; doi:10.1128/mBio.00760-21)

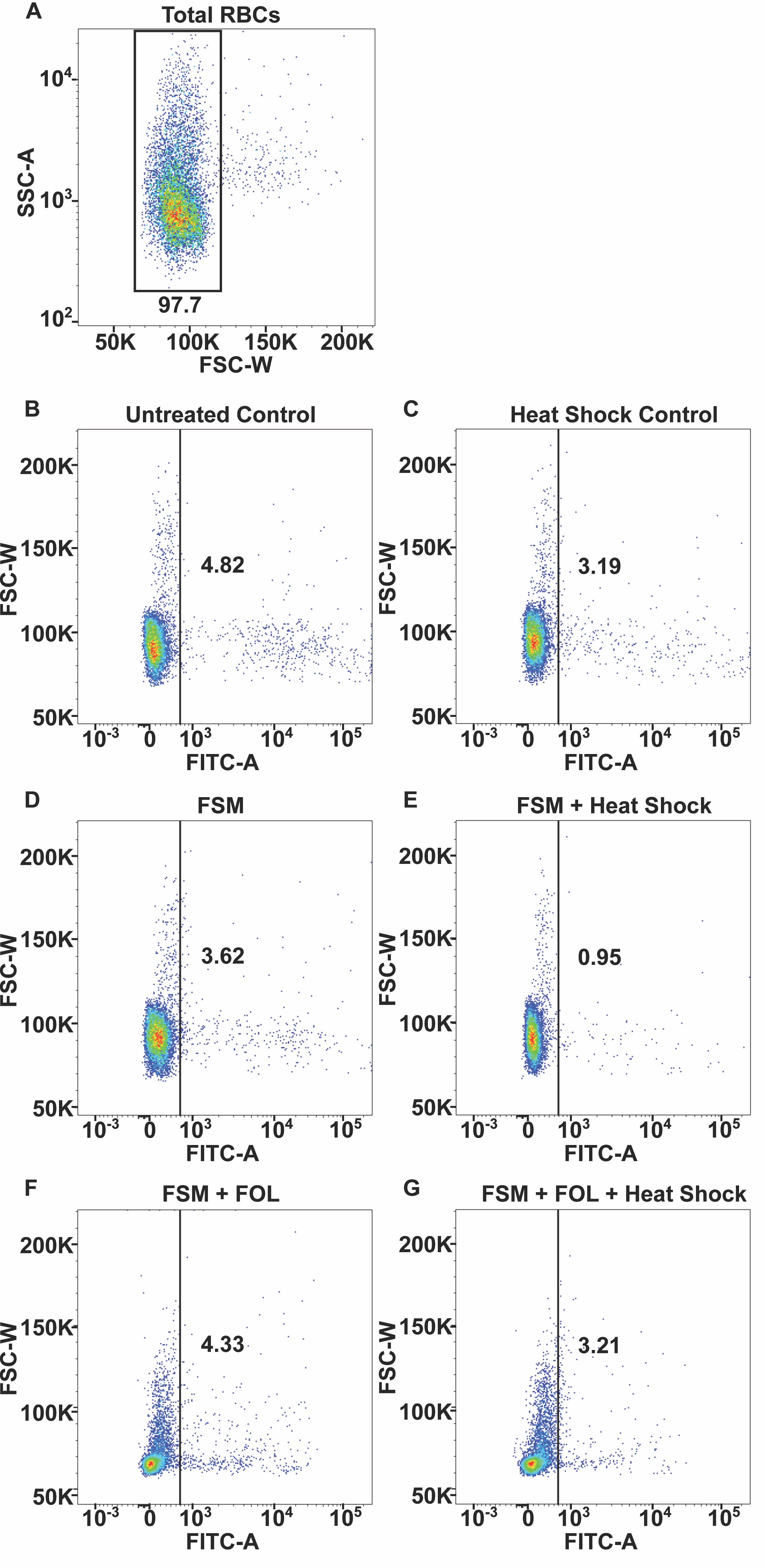

Supplement: FIG S1 [file mbio.00760-21-sf001.jpg]

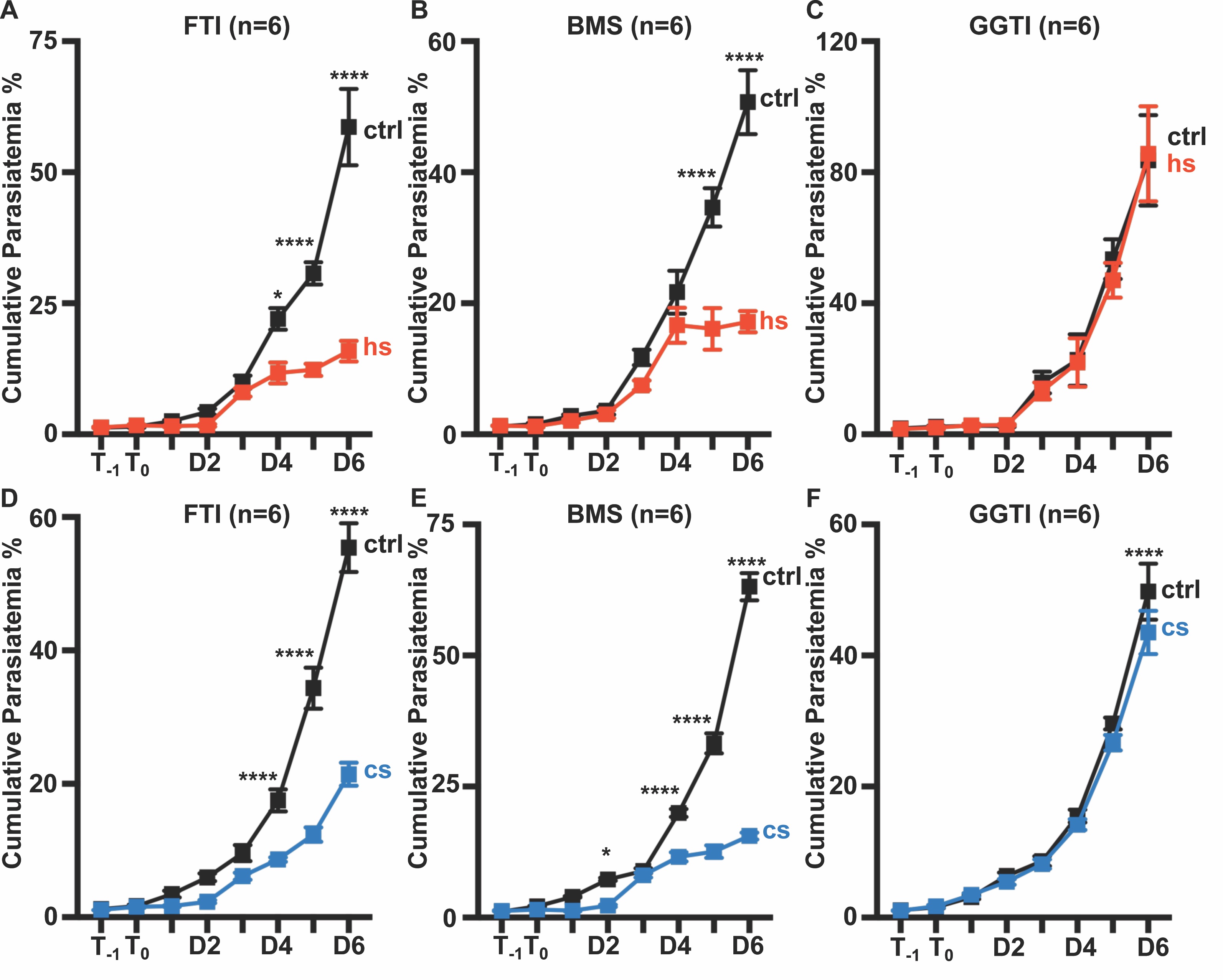

Supplement: FIG S2 [file mbio.00760-21-sf002.jpg]

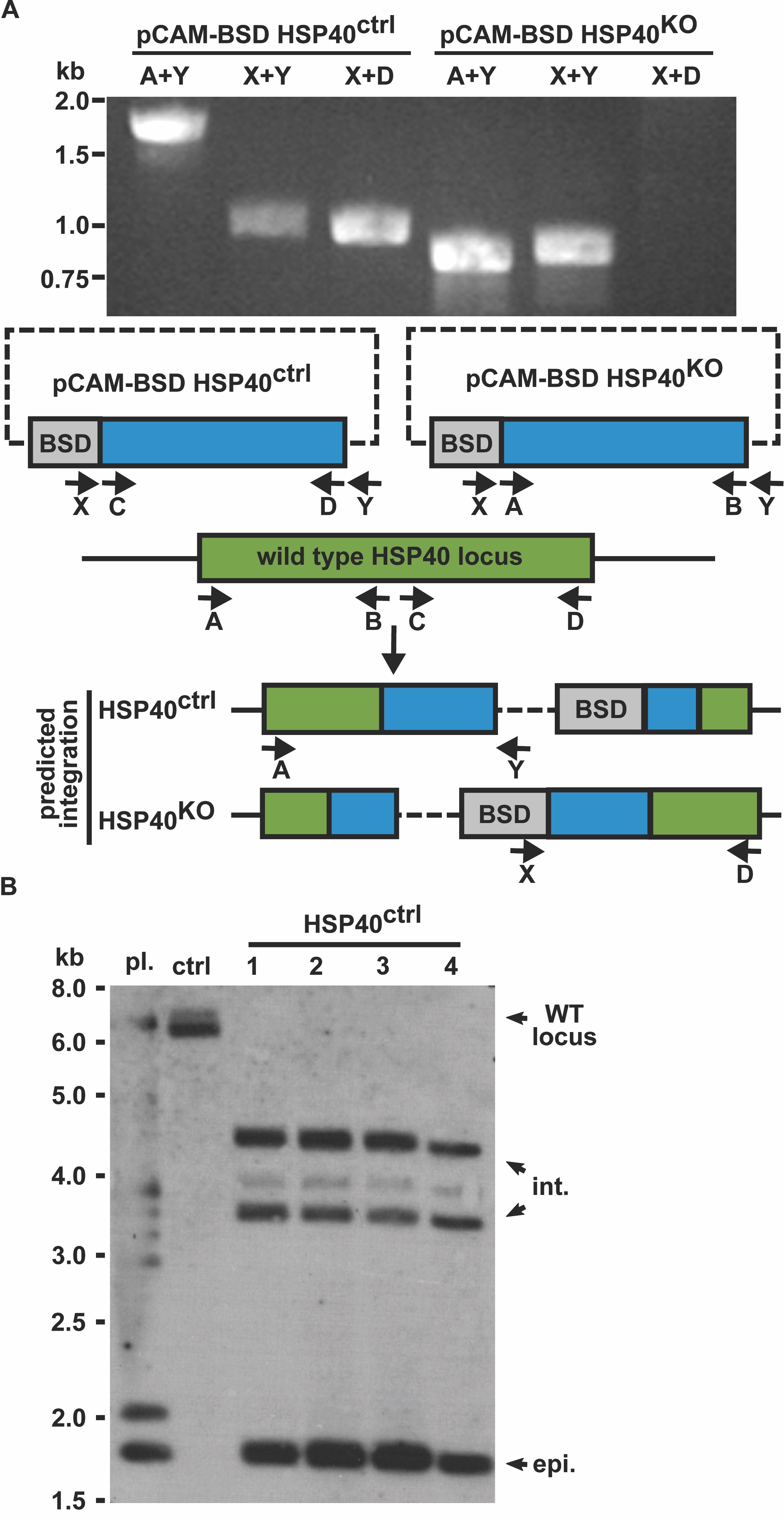

Supplement: FIG S3 [file mbio.00760-21-sf003.jpg]

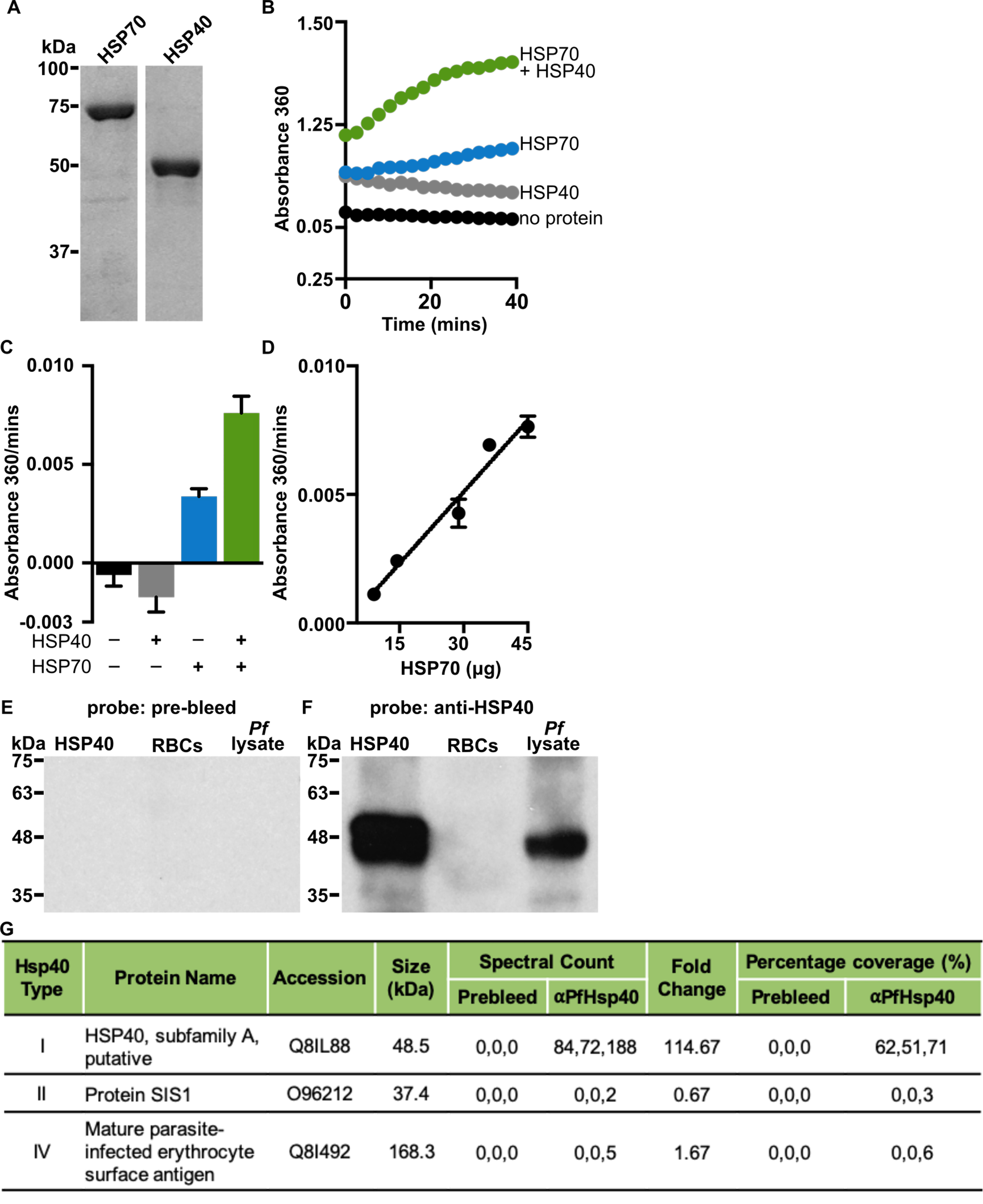

Supplement: FIG S4 [file mbio.00760-21-sf004.jpg]

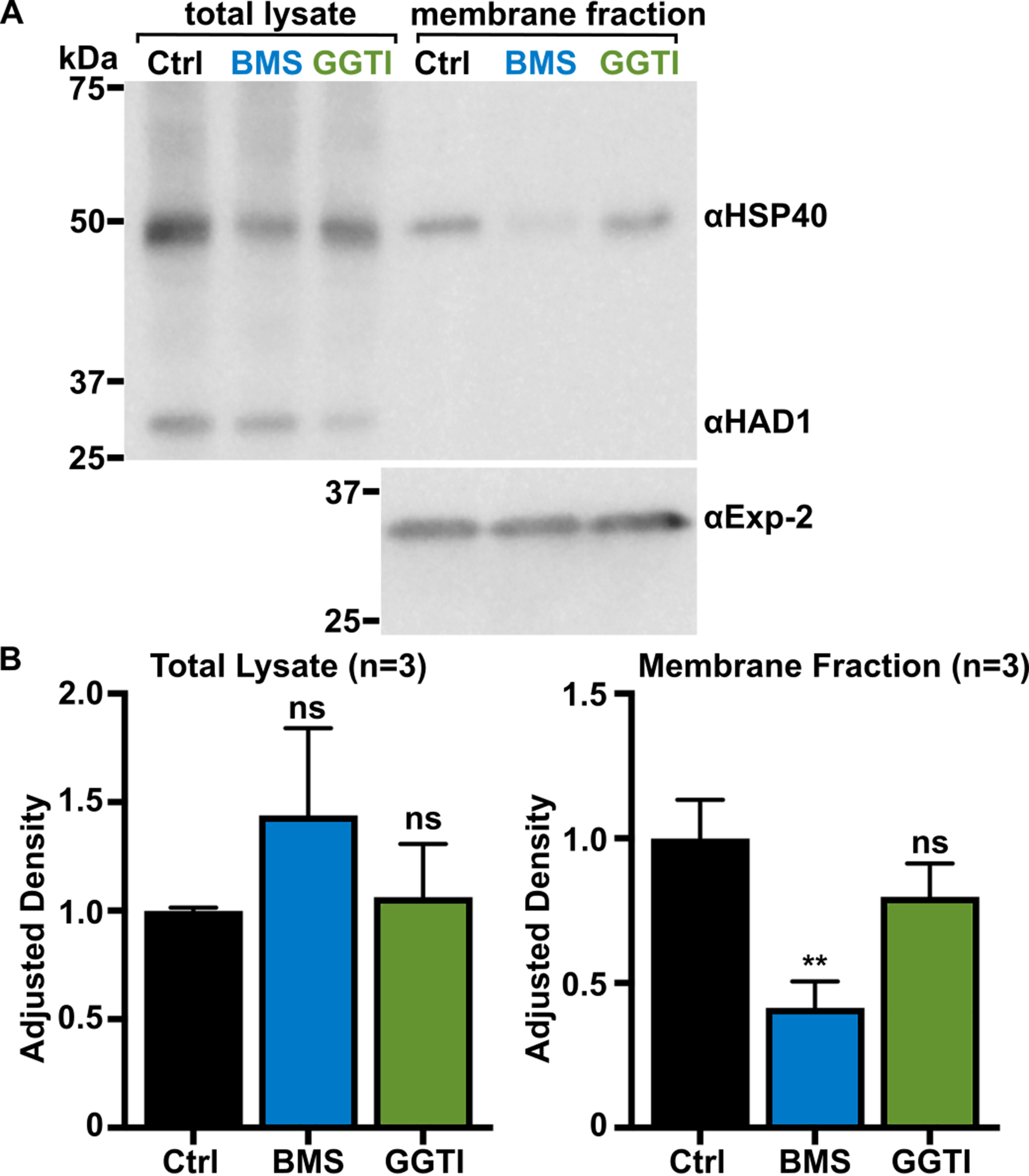

Supplement: FIG S5 [file mbio.00760-21-sf005.jpg]

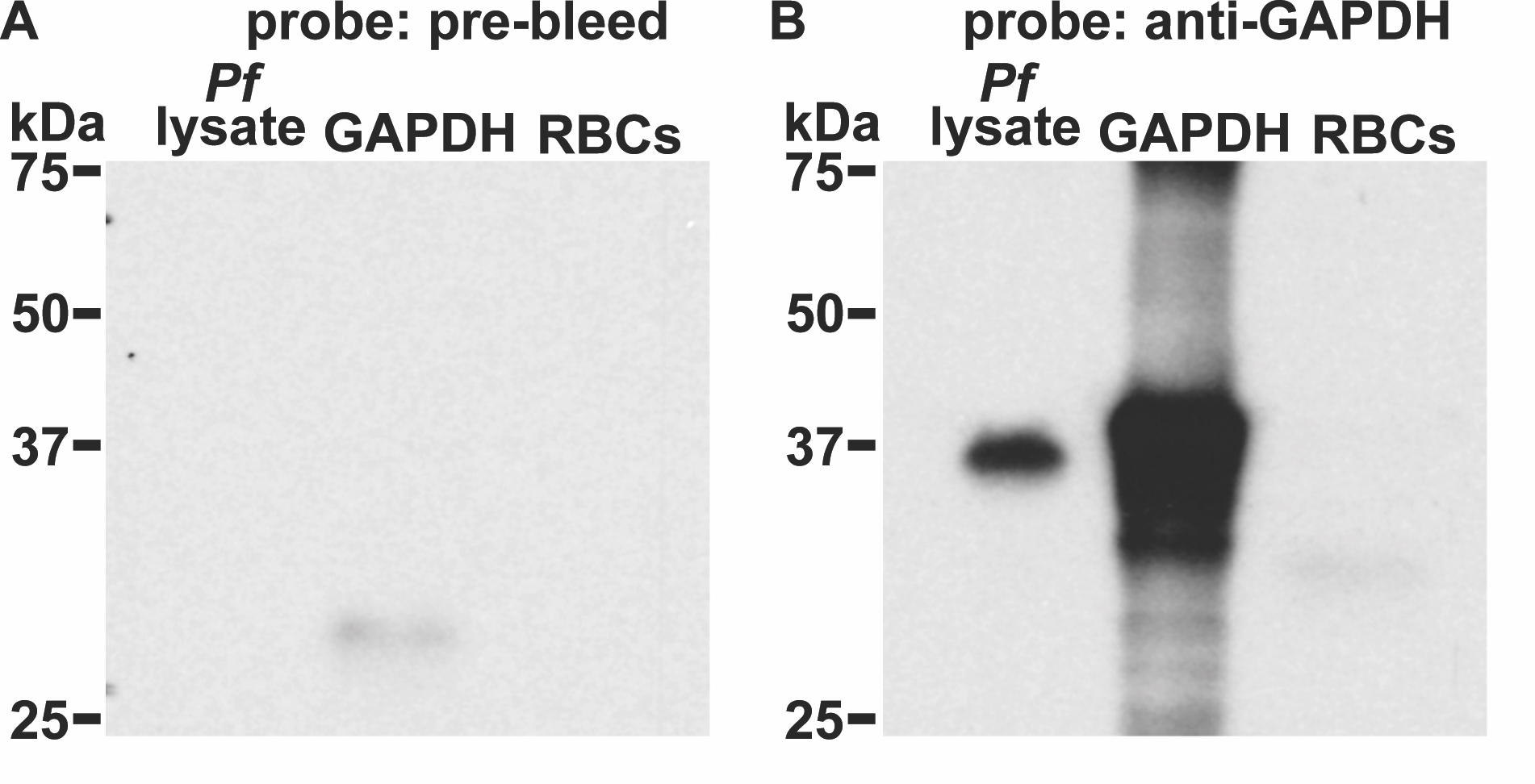

Supplement: FIG S6 [file mbio.00760-21-sf006.jpg]
