# Supplementary material for: Protein Prenylation and Hsp40 in Thermotolerance of Plasmodium falciparum Malaria Parasites
Source: mBio. 2021 Jun 29;12(3):e00760-21. doi: 10.1128/mBio.00760-21 (PMC8262983; doi:10.1128/mBio.00760-21)
Supplement: TABLE S1 [file mbio.00760-21-st001.docx]

| **Protein IDs** | **Gene ID** | **Protein annotation** |
| --- | --- | --- |
| C0H4Y6 | PF3D7_0827900 | Protein disulfide-isomerase |
| C0H5C2 | PF3D7_1317800 | 40S ribosomal protein S19 |
| C6KTA4 | PF3D7_0626800 | Pyruvate kinase |
| O97248 | PF3D7_0306900 | 40S ribosomal protein S23, putative |
| Q6ZLZ9 | PF3D7_0903700 | Tubulin alpha chain |
| Q76NM3 | PF3D7_1324900 | L-lactate dehydrogenase |
| Q7KQL5 | PF3D7_1008700 | Tubulin beta chain |
| Q7KQL8 | PF3D7_1457200 | Thioredoxin (Trx) |
| Q8I0V4 | PF3D7_1222300 | Endoplasmin, putative (GRP94/HSP90) |
| Q8I298 | PF3D7_0104200 | StAR-related lipid transfer protein |
| Q8I2Q0 | PF3D7_0925900 | Parasitophorous vacuolar protein 5, putative |
| Q8I3U6 | PF3D7_0516200 | 40S ribosomal protein S11 |
| Q8I431 | PF3D7_0507100 | 60S ribosomal protein L4 |
| Q8IBA0 | PF3D7_0826700 | Receptor for activated c kinase (RACK1) |
| Q8IBN5 | PF3D7_0721600 | 40S ribosomal protein S5, putative |
| Q8IDR9 | PF3D7_1342000 | 40S ribosomal protein S6 |
| Q8II24 | PF3D7_1134000 | Heat shock protein 70 (HSP70-3) |
| Q8IJX8 | PF3D7_1006200 | DNA/RNA-binding protein Alba 3 |
| Q8IKF0 | PF3D7_1468700 | Eukaryotic initiation factor 4A |
| Q8IKH8 | PF3D7_1465900 | 40S ribosomal protein S3 |
| Q8IKL9 | PF3D7_1461300 | 40S ribosomal protein S28e, putative |
| Q8IL88 | PF3D7_1437900 | HSP40, subfamily A |
| Q8ILE8 | PF3D7_1431700 | 60S ribosomal protein L14, putative |
| Q8IM15 | PF3D7_1408100 | Plasmepsin III |

**Supplemental Table 1. Candidate HSP40 interacting proteins.** Proteins that immunoprecipitated with anti-HSP40 (PF3D7_1437900) antisera in three independent experiments, but not with negative control pre-bleed sera from the same animals, were identified by mass spectrometry. List is limited to those proteins that associated with Hsp40 in the absence of drug treatment.
