# Supplementary material for: Protein Prenylation and Hsp40 in Thermotolerance of Plasmodium falciparum Malaria Parasites
Source: mBio. 2021 Jun 29;12(3):e00760-21. doi: 10.1128/mBio.00760-21 (PMC8262983; doi:10.1128/mBio.00760-21)
Supplement: TABLE S2 [file mbio.00760-21-st002.docx]

| **Protein IDs** | **Gene ID** | **Protein Annotation** |
| --- | --- | --- |
| Q8IKK7 | PF3D7_1462800 | Glyceraldehyde-3-phosphate dehydrogenase (EC 1.2.1.12) |
| Q8IIA2 | PF3D7_1126200 | 40S ribosomal protein S18, putative |
| Q8IFP2 | PF3D7_0422400 | 40S ribosomal protein S19 |
| ACT1 | PF3D7_1246200 | Actin-1 (Actin I) |
| Q8I0U8 | PF3D7_0930300 | Merozoite surface antigens (Merozoite surface protein 1) (PMMSA) |
| Q8IKF0 | PF3D7_1468700 | Eukaryotic initiation factor 4A (EC 3.6.4.12) |
| Q76NM3 | PF3D7_1324900 | L-lactate dehydrogenase |
| Q8IKH8 | PF3D7_1465900 | 40S ribosomal protein S3 |
| Q8IDR9 | PF3D7_1342000 | 40S ribosomal protein S6 |
| Q8IBN5 | PF3D7_0721600 | 40S ribosomal protein S5, putative |
| Q6ZLZ9 | PF3D7_0903700 | Tubulin alpha chain |
| Q8IC05 | PF3D7_0708400 | Heat shock protein 90 |
| C0H5C2 | PF3D7_1317800 | 40S ribosomal protein S19 |
| Q8IM10 | PF3D7_1408600 | 40S ribosomal protein S8 |
| Q8IAX5 | PF3D7_0813900 | 40S ribosomal protein S16, putative |
| Q8I2Q0 | PF3D7_0925900 | Parasitophorous vacuolar protein 5, putative |
| TBB | PF3D7_1008700 | Tubulin beta chain (Beta-tubulin) |
| Q8II62 | PF3D7_1130100 | 60S ribosomal protein L38 |
| Q8I2X3 | PF3D7_0918000 | Acid phosphatase (EC 3.1.3.2) |
| O97248 | PF3D7_0306900 | 40S ribosomal protein S23, putative |
| Q8I0P6 | PF3D7_1357000 | Elongation factor 1-alpha |
| Q8ILL3 | PF3D7_1424100 | 60S ribosomal protein L5, putative |
| Q8I3U6 | PF3D7_0516200 | 40S ribosomal subunit protein S11 |
| Q8I3R0 | PF3D7_0520000 | 40S ribosomal protein S9, putative |
| Q7KQK6 | PF3D7_1117700 | GTP-binding nuclear protein |
| Q8IJX8 | PF3D7_1006200 | DNA/RNA-binding protein Alba 3 |
| Q8I2X4 | PF3D7_0917900 | Heat shock protein 70 |
| Q8IM15 | PF3D7_1408100 | Plasmepsin III (EC 3.4.23.-) |
| Q7K6A4 | PF3D7_0922200 | S-adenosylmethionine synthase (EC 2.5.1.6) |
| C6KTA4 | PF3D7_0626800 | Pyruvate kinase (EC 2.7.1.40) |
| OAT | PF3D7_0608800 | Ornithine aminotransferase (EC 2.6.1.13) (Ornithine--oxo-acid aminotransferase) |
| Q8IKL9 | PF3D7_1461300 | 40S ribosomal protein S28e, putative |
| ENO | PF3D7_1015900 | Enolase (EC 4.2.1.11) (2-phospho-D-glycerate hydro-lyase) (2-phosphoglycerate dehydratase) |
| Q8IEN2 | PF3D7_1308300 | 40S ribosomal protein S27 |
| Q8IHU0 | PF3D7_1142500 | 60S ribosomal protein L28 |
| C0H4Y6 | PF3D7_0827900 | Protein disulfide-isomerase (EC 5.3.4.1) |

**Supplemental Table S2. Gene/protein IDs and annotations of candidate HSP40-interacting proteins.** Proteins indicated are those whose association changed on treatment with fosmidomycin or farnesyl transferase inhibition.
